# Beyond Overlap: Development and Validation of the Inclusion of AI in Self (IAIS) Scale and Its Moderating Role in the Relationship Between AI Identity and AI Threat

**DOI:** 10.3390/jintelligence14100239

**Published:** 2026-10-02

**Authors:** Jiali He, Hanmi Lee, Xiang Yao

**Affiliations:** School of Psychological and Cognitive Sciences, Key Laboratory of Machine Perception (Ministry of Education), Beijing Key Laboratory of Brain-Computer Interface and Mental Health Modulation, Peking University, Beijing 100871, China; 2601110857@stu.pku.edu.cn (J.H.); 2501110742@stu.pku.edu.cn (H.L.)

**Keywords:** inclusion of AI in self, self-expansion theory, scale validation, AI threat, AI literacy, longitudinal study

## Abstract

Artificial intelligence (AI) is becoming increasingly integrated into people’s self-concepts, yet a psychometrically robust multi-item pictorial instrument for assessing the inclusion of AI in self (IAIS) is lacking. Drawing on self-expansion theory and the AI-mediated communication (AI-MC) model, we developed and validated the IAIS scale. The IAIS comprises four pictorial items capturing overlap, distance, centrality, and size, designed as indicators of a single dimension. In Study 1, Chinese university students (*N* = 195, 56.9% male) were recruited through campus online platforms using convenience sampling. We conducted exploratory and confirmatory factor analyses (EFA/CFA), examined reliability and convergent, discriminant, and criterion-related validity (the last via Steiger’s Z tests), and assessed gender invariance with multigroup CFA. In Study 2, the scale was cross-validated in a sample of Chinese employees (*N* = 513, 38.4% male) recruited through the Credamo online research platform. In Study 3, a mixed student–employee sample (*N* = 240, 43.3% male) completed a two-wave longitudinal survey with a 15-day interval. The moderating role of IAIS in the relationship between AI identity and AI threat was tested with PROCESS Model 1 (5000 bootstrap resamples). Results supported a replicable single-factor structure, good reliability, and convergent, discriminant, and criterion-related validity. IAIS significantly moderated the association between AI identity and AI threat. We conclude that IAIS is a psychometrically sound instrument for assessing the inclusion of AI in self, and that the negative association between AI identity and AI threat is stronger among individuals who include AI more deeply in the self.

## 1. Introduction

As generative artificial intelligence (AI) evolves from an assistive tool into a work partner capable of independently executing knowledge-intensive tasks, the employee–technology relationship is changing. One way to understand this shift is through self-expansion theory, which argues that humans have a basic drive to bring others into the self, a process known as inclusion of other in the self (IOS). Within close relationships, people absorb a partner’s resources, perspectives, and identities into their self-concept, which in turn expands the boundaries of the self and enhances potential efficacy (Aron et al., 1991, 1992; Aron & Aron, 1986). Building on this idea, researchers have extended the IOS logic to the technology domain and proposed the concept of information technology (IT) identity, defined as the extent to which individuals view IT use as an integral component of their self-concept. IT identity comprises three core dimensions: relatedness, emotional energy, and dependence (Carter & Grover, 2015). Recently, the same logic has been applied to AI, producing constructs such as AI identity (Mirbabaie et al., 2022). However, AI identity primarily reflects the affective attachment and behavioral dependence that follow from integrating AI into one’s self-concept; that is, it captures downstream outcomes rather than the inclusion process itself (Ashraf et al., 2026). Consequently, an exclusive focus on outcomes leaves unclear whether elevated AI identity stems from deep self-concept reconstruction or merely from superficial emotional dependence and behavioral inertia. Compared with outcome-focused research, process-oriented research may be better suited to uncovering the “dark side” of AI (e.g., AI threat).

Although existing studies have examined the cognitive process of the inclusion of AI in self (IAIS) using the IOS scale (Huang et al., 2025; Liang & Banks, 2025), important gaps remain. First, at the theoretical level, prior literature has yet to explicitly propose the inclusion of AI in self as a psychologically meaningful construct. Although traditional extended-self theory recognizes individuals’ basic tendency to incorporate possessions into self-representation (Belk, 1988), digital technologies have changed the forms self-expansion takes, making them more complex and varied (Belk, 2013). In interactions with AI, such expansion may manifest as functional augmentation (e.g., enhanced task performance and efficiency), cognitive scaffolding (e.g., externalized thinking through retrieval–generation processes), or, in specific situations, relational experiences (e.g., companionship, attachment, and anthropomorphism) (Campbell et al., 2025). Symbiotic technology theory further posits that AI has evolved into a symbiotic entity possessing reflexivity, emotional resonance, and co-evolutionary capabilities, such that humans and AI form a bidirectional feedback loop and maintain a dynamic balance in a shared cognitive field (Van Lu, 2025). This contrasts with the unidirectional extension logic of “inclusion of other in self” assumed by the traditional IOS framework, which is grounded in interpersonal intimacy.

Second, at the measurement level, existing measures share the inherent limitations of single-item designs. The IOS scale is a single-item pictorial measure suited to quickly capturing general intimacy or overall self–other fusion (Aron et al., 1992). Given that AI can expand the self through several pathways (Campbell et al., 2025), a single item provides insufficient content coverage of AI self-expansion. In addition, a single item does not allow internal consistency reliability to be estimated, which limits rigorous psychometric evaluation (Allen et al., 2022; Cronbach, 1951). Single items are also more susceptible to random influences such as transient participant states and item misinterpretation, which can increase measurement error and reduce stability (Diamantopoulos et al., 2012). Moreover, single items are poorly suited to complex statistical analyses, which hinders empirical testing of AI-mediated self-expansion theory, cross-contextual validation, and cross-study comparisons (Iacobucci, 2010). The IAIS scale moves beyond the single overlap metaphor by capturing four complementary spatial facets of self–AI representation (overlap, distance, centrality, and size), an approach adapted from the Extended Inclusion of Nature in Self scale (EINS; Martin & Czellar, 2016). Whereas the overlap item indexes only the degree of self–other fusion emphasized in the classic IOS tradition (Aron et al., 1992), the additional metaphors of distance, centrality, and size capture how close AI feels to the self, how centrally AI is positioned within identity, and how much relative weight it carries in self-representation.

Third, at the level of mechanism, existing research has yet to systematically link inclusion of AI in self to negative consequences in organizational contexts (e.g., AI threat). Empirical findings regarding the relationship between AI identity and AI threat have been inconsistent (Ashraf et al., 2026; Mirbabaie et al., 2022). One likely reason is that AI identity, as an outcome state, does not reflect the degree of cognitive integration between AI and the self. By contrast, inclusion of AI in self, as an indicator of integration depth, may help explain these inconsistent findings. However, research has not yet systematically tested whether inclusion of AI in self directly predicts AI threat perception or acts as a boundary condition (i.e., a moderator).

Accordingly, the present study aims to clarify the concept and factor structure of IAIS and to develop a psychometrically robust measure of this construct. Following the scale development approach of the EINS (Martin & Czellar, 2016), we developed and validated the IAIS scale through a series of empirical studies, evaluating its factor structure, reliability, convergent validity, discriminant validity, criterion-related validity, and psychometric advantages relative to the traditional single-item measure. In doing so, we tested whether IAIS could be reliably and validly represented as a unidimensional construct using a multi-metaphor pictorial format. Furthermore, in a short-term longitudinal study, we examined whether IAIS functions as a boundary condition that moderates the association between AI identity and perceived AI threat. Together, these studies provide a standardized measurement tool for future research on AI-related self-expansion and clarify the conceptual and functional role of IAIS in how individuals relate to AI.

### 1.1. Self-Expansion Theory and Inclusion of the Self

Self-expansion theory posits a core motivation to enlarge the self-concept in order to increase potential efficacy, defined as the capacity to achieve goals more effectively by acquiring new resources, capabilities, perspectives, and identities (Aron & Aron, 1997). In the context of intimate relationships, a central socio-cognitive pathway for realizing this motivation is the inclusion of other in the self (IOS). When an external object (typically a romantic partner) consistently supports goal attainment and capability growth through interaction, the individual cognitively increases the perceived overlap between the self and that object, and to some extent experiences the other’s resources, capabilities, identities, and perspectives as “part of the self,” resulting in partial fusion of self–other boundaries (Aron et al., 1991). Building on this mechanism, researchers developed the classic single-item IOS scale, which presents seven sets of self–other Venn diagrams (circles) ranging from “almost non-overlapping” to “highly overlapping” and asks participants to select the figure that best represents their relationship with another person, thereby measuring intimacy between the self and others (Aron et al., 1992). Subsequently, the pictorial metaphor of the IOS was widely extended to nonhuman objects or more macro-level entities (e.g., ingroups, communities, nature) to intuitively assess the degree of “inclusion in the self” (Martin & Czellar, 2016; Mashek et al., 2007; Tropp & Wright, 2001).

This successive transfer of the pictorial format across human others, nature, ingroups, and communities reflects a general cognitive mechanism of self–other representation. The self’s boundary is flexible and can be reorganized, such that potentially any entity that reliably supports goal attainment can be incorporated into the self-structure irrespective of whether it is human or nonhuman (Aron et al., 1991). Within this object-independent mechanism, the four spatial metaphors of overlap, distance, centrality, and size serve as four reflective indicators of a single construct, the inclusion of the entity in the self. Overlap captures the permeability of the self’s boundary, distance captures the entity’s proximity to the self, centrality captures its position at the core or periphery of the self, and size captures the relative weight it carries in self-representation. The facets captured by these indicators are abstract relational properties of the self-structure rather than features specific to any particular object, which is why the pictorial format is transferable across qualitatively different targets (Martin & Czellar, 2016).

In extending “inclusion in the self” from interpersonal targets to broader external entities, researchers used the “self–nature relationship” as an exemplar and proposed a cognitive definition of “inclusion of nature in the self,” establishing the single-item pictorial measurement tradition of the Inclusion of Nature in Self (INS) scale (Schultz, 2002). However, single-item pictorial measures are inherently limited psychometrically (e.g., difficulty estimating measurement error and limited coverage of structurally complex constructs). Therefore, Martin and Czellar (2016) proposed and validated the Extended Inclusion of Nature in Self (EINS) scale. This scale preserves the spatial pictorial representation of “self–object” relationships but expands the single “overlap” metaphor into four spatial-metaphor items to improve reliability, validity, and applicability. The four metaphors are as follows. (1) Overlap corresponds to the original single-item INS scale (Schultz, 2002) and reflects the degree of fusion between the self and the object. This item primarily measures the sense of connection, that is, the degree to which individuals cognitively include nature within the boundaries of the self. (2) Distance ranges from separation (distant) to proximity (overlapping) and reflects the psychological closeness between the self and the object. This item draws on the metaphor of physical closeness: perceiving spatial proximity can activate and intensify individuals’ affective experiences of intimacy (Williams & Bargh, 2008). (3) Centrality measures how central nature is within the self-structure through changes in the position of the nature circle in a cross-coordinate system (from peripheral offset to central overlap); it draws on the importance-as-central-position metaphor found in concepts such as work centrality (Paullay et al., 1994). (4) Size draws on the importance-as-size metaphor (Bruner & Postman, 1958) and reflects, through changes in the absolute size of the circle (from extremely small to extremely large), how much relative importance individuals perceive nature to have in the self-structure.

### 1.2. Theoretical Foundations of Inclusion of AI in Self

Extending this theoretical lineage to the AI context, and building on extended-self theory and the three-part framework of functional, ontological, and anthropomorphic self-extension (Belk, 1988; Park & Kaye, 2019), the AI-mediated communication (AI-MC) model proposes that AI can expand the self through three pathways: functional self-extension, anthropomorphic self-extension, and ontological self-extension (Campbell et al., 2025).

In functional self-extension, AI typically enters the individual’s goal-pursuit system as an “environmental scaffold” or “external brain.” As an external cognitive resource available for immediate deployment, it forms a high-frequency, low-friction coupling loop with the individual, thereby expanding the person’s problem-solving space, information-processing bandwidth, and range of feasible actions. This pathway aligns with extended mind theory, which posits that when an external system is stable, accessible, and reliable and is tightly and bidirectionally coupled with the agent, it can become part of the cognitive process, forming a “human–AI” hybrid thinking system (Clark, 2025; Clark & Chalmers, 1998). Such coupling thus produces functional self-extension.

In anthropomorphic self-extension, the self-expansion triggered by AI first appears as individuals come to view AI as a “relational other,” thereby generating subjective self–AI intimacy at the experiential level. For example, users treat AI’s understanding, responsiveness, and “emotional presence” as relational resources for regulating loneliness and seeking support and validation; through sustained interaction, they also incorporate its viewpoints and feedback into self-evaluation and action selection and become more prone to emotional attachment to AI (Pentina et al., 2023). A 21-day randomized controlled longitudinal study found that the level of AI anthropomorphism mediates the impact of human–AI interaction on social life (Guingrich & Graziano, 2025). Large-sample experiments have also shown that individuals higher in anthropomorphism can experience socioemotional connection through AI companionship (Folk et al., 2025).

In ontological self-extension, AI’s influence on the self goes beyond its roles as a “relational other” and a “cognitive tool” and touches on the process of self-construction, that is, “who am I and how do I become myself.” When AI begins to predict and intercept human intentions, expressions, and choices ever more frequently and preemptively, the individual’s relationship with the self may be reorganized into a “self–world relationship” shaped jointly by algorithmic feedback and generative capabilities (Schiller et al., 2025). For instance, the algorithmic self does not passively reflect the self but actively shapes the individual’s understanding of their own preferences, emotions, and motivations through continuous recommendations, categorizations, feedback, and mirroring (Camacho, 2026; Joseph, 2025); in doing so, it reaches core areas of the individual’s self-concept.

Although functional, anthropomorphic, and ontological self-extension routes describe distinct ways in which AI gains access to the self, these routes are theorized to facilitate one another and to co-occur rather than to operate independently. They therefore jointly constitute a single latent construct: the inclusion of AI in self. Consistent with this, the four spatial metaphors used to operationalize this construct are not four independent dimensions but four reflective indicators of a single underlying process (Bollen & Lennox, 1991; Jarvis et al., 2003). Overlap, distance, centrality, and size each capture a complementary facet of how far AI has been integrated into the self-structure, and they covary because they share this common core. This treatment follows directly from the EINS precedent, in which the four spatial metaphors were likewise shown to load on a single factor rather than on separate dimensions (Martin & Czellar, 2016). More broadly, in stable collaboration with AI, individuals incorporate AI’s resources, capabilities, and perspectives into their self-representation and action systems to enhance potential efficacy. This process encompasses functional, anthropomorphic, and ontological forms of inclusion, which suggests that inclusion in the self is a general mechanism that operates across objects and contexts (Aron et al., 1991; Belk, 1988; Clark & Chalmers, 1998; Hutchins, 1995).

### 1.3. Overlap, Distance, Centrality, and Size

Given this theoretical integration, adapting the multi-metaphor approach of the EINS to construct IAIS enables more comprehensive coverage of the structural complexity of AI self-expansion at both conceptual and measurement levels. Specifically, IAIS uses the four spatial metaphors of the EINS as anchors and defines each of them for the AI context.

Overlap reflects the degree of sharing and fusion between the self and AI in cognitive processing, knowledge, and decision-making. It indicates how deeply AI, as an “environmental scaffold,” is intertwined with the individual’s thinking in functional self-extension. When individuals perceive high overlap between their own thinking and AI, they regard AI’s capabilities as extensions and scaffolds of their own capabilities, which enhances their confidence and efficacy judgments about completing complex tasks.

Distance reflects the psychological closeness and emotional accessibility between the individual and AI. It captures the subjective experience in anthropomorphic self-extension in which individuals perceive AI as a relational other and develop intimacy and emotional attachment. The smaller the psychological distance, the more individuals tend to perceive AI as a trustworthy, reliable relational other, which may in turn produce typical attachment responses such as proximity seeking, safe-haven seeking, and separation anxiety.

Centrality reflects the core position of AI in the individual’s self-concept and identity narrative. It relates to the fundamental question of “who am I” in ontological self-extension, that is, whether AI becomes a key reference point for self-understanding, self-expression, and self-continuity. When AI becomes central to how individuals understand who they are and enters their self-continuity and identity narrative, it may promote the formation of an identity structure centered on technology use and human–AI symbiosis.

Size reflects the relative proportion AI occupies in the individual’s self-representation, indicating the importance of AI as a cognitive resource and identity element in the self-structure. The larger the proportion AI occupies in the self-structure, the more the individual outsources cognitive, emotional, and behavioral functions to AI, and the more difficult it becomes to function without AI, which manifests as stable dependence in behavior and emotion.

Consistent with the EINS precedent (Martin & Czellar, 2016), these four spatial metaphors jointly represent complementary facets of AI self-expansion as proposed by the AI-MC model (see Table 1) and serve as four items of a single construct, the inclusion of AI in self, rather than as four distinct dimensions.

The inclusion of AI in self is related to, yet conceptually distinct from, several adjacent constructs. AI identity refers to the extent to which individuals regard their use of or collaboration with AI as integral to their self-concept (Carter & Grover, 2015; Mirbabaie et al., 2022). As an outcome state, it reflects the affective and dependence-related consequences of incorporation, whereas IAIS captures the incorporation process itself: one may identify strongly as an AI collaborator without experiencing AI as part of the self. AI attachment denotes the affective bond directed toward AI as a relational other, and it indexes the anthropomorphic rather than the ontological pathway (Yang & Oshio, 2025). Anthropomorphism, or the attribution of human-like qualities to AI, is a factor that can facilitate self-incorporation (Epley et al., 2007). Crucially, however, anthropomorphism and IAIS can each occur without the other. Extended cognition refers to AI operating as an external cognitive scaffold that need not enter the self-concept (Clark & Chalmers, 1998). Technological dependence is a behavioral reliance on AI that may arise from habit or inertia without deep self-incorporation (Zhang et al., 2025). IAIS therefore cannot be reduced to relational closeness, functional extension, behavioral reliance, or the mere attribution of human-like qualities to AI. It specifically captures the incorporation of AI into the perceived boundaries and organization of the self, that is, the extent to which AI is experienced as overlapping with, proximal to, central within, or occupying a large part of one’s self-representation.

### 1.4. The Moderating Role of IAIS in the Relationship Between AI Identity and AI Threat

Prior research indicates that AI identity negatively predicts AI threat; that is, the stronger individuals’ emotional attachment to and identity integration with AI, the lower their perceived AI replacement anxiety and occupational identity threat (Mirbabaie et al., 2022). However, recent research has found that the direct effect of AI identity on identity threat is nonsignificant (Ashraf et al., 2026), and the boundary conditions and stability of this effect remain unclear. The inconsistency across these findings may arise because existing research largely treats AI identity as a simple state and does not distinguish the different cognitive processes underlying it. Specifically, high AI identity may derive from deep cognitive restructuring in which individuals incorporate AI into self-schemas through self-expansion mechanisms, or it may merely reflect a superficial attachment state driven by emotional energy and behavioral dependence (Alabed et al., 2022; Campbell et al., 2025; Carter & Grover, 2015). These two pathways may relate to AI threat in different ways, and IAIS may serve as the moderator that distinguishes between them.

From the perspective of inclusion of AI in self, deep self–AI inclusion involves a cognitive restructuring process in which individuals incorporate AI into self-schemas, which manifests as active expansion of capability boundaries and redefinition of occupational roles (Alabed et al., 2022; Aron & Aron, 1986). Such deep inclusion may help individuals reinterpret AI’s enhanced autonomy as an expansion of the shared capabilities of humans and AI and thereby buffer AI threat (Ashraf et al., 2026). In contrast, AI identity may remain superficial, driven by emotional energy and behavioral dependence, and manifest as emotional attachment to AI and habitual use (Carter & Grover, 2015; Skjuve et al., 2021). When AI demonstrates autonomous decision-making capabilities beyond expectations, such superficial identity, unaccompanied by deeper schema updating, may not only fail to buffer threat but also amplify occupational identity threat by eroding the sense of human distinctiveness through over-identification (Ashraf et al., 2026). Therefore, we expect IAIS to strengthen the negative association between AI identity and AI threat: the higher IAIS, the more strongly AI identity should be negatively associated with AI threat.

### 1.5. Current Study

In sum, grounded in self-expansion theory and the AI-MC model, the present study extends the classic pictorial measurement paradigm of inclusion in the self to AI-mediated contexts by developing and validating the IAIS scale, a measure suited to AI use and human–AI collaboration. In doing so, it expands the traditional single-item representation of inclusion in the self into a four-item pictorial scale covering overlap, distance, centrality, and size. This approach clarifies how the inclusion of AI in the self is cognitively represented and addresses the inability of single-item measures to reveal construct structure. Following established scale development guidelines, this study examines the reliability, structure, and validity of IAIS to provide a psychometrically sound instrument for this domain.

Furthermore, this study tests whether IAIS is empirically distinct from related constructs and whether it acts as a boundary condition in the relationship between AI identity and AI threat. This provides a more precise theoretical and measurement basis for understanding how human–AI interaction in AI-mediated contexts shapes individuals’ threat perception. The following hypotheses are proposed:

**Hypothesis** **1.** *IAIS demonstrates good reliability and validity among university students.*

**Hypothesis** **2.** *IAIS demonstrates good reliability and validity among employees.*

**Hypothesis** **3.** *IAIS moderates the relationship between AI identity and AI threat perception, such that the negative association is stronger when IAIS is higher.*

## 2. Study 1

The primary objective of Study 1 was to examine the psychometric properties of IAIS and preliminarily evaluate its measurement performance by comparing it with the single-item measure. Specifically, AI identity, AI attachment, AI literacy, AI self-efficacy, and AI chatbot dependence served as criterion variables. Criterion-related validity was assessed by comparing correlation coefficients with Steiger’s Z tests, and discriminant validity was assessed with the Fornell–Larcker criterion and the HTMT ratio.

### 2.1. Participants and Procedure

Sample size was determined following established guidelines for scale development and validation. For factor analysis, the common rule of thumb is a participant-to-item ratio of 5:1 to 10:1, but this alone is insufficient for short scales (Comrey & Lee, 1992). CFA guidelines recommend a minimum of 100 participants even for scales with few items. The target size for Sample 1 was therefore set at 200, meeting both the ratio guideline and the absolute minimum for factor-analytic stability, while allowing for exclusion of invalid responses. For test–retest reliability, a sample of at least 30 participants is considered adequate to estimate the intraclass correlation coefficient (ICC) with acceptable precision (Koo & Li, 2016).

Study 1 involved two independent samples recruited for distinct analytic purposes. Sample 1 was used for scale development and validation. The inclusion criteria were (a) being a currently enrolled university student in China, (b) being aged 18 years or older, (c) being able to read and complete the Chinese-language questionnaire, and (d) having prior experience using AI. Participants were recruited from Chinese university campus social groups via an online survey link; 200 students initially participated. After excluding five cases with excessively short response times (less than 60 s), the final dataset comprised 195 participants (*M*_age_ = 22.70, *SD* = 1.87; 56.9% male). With respect to educational attainment, 24 participants (12.3%) were vocational college students, 161 (82.6%) were undergraduates, and 10 (5.1%) were graduate students or above. Upon completion, each participant received fixed monetary compensation. Data from Sample 1 were used for exploratory/confirmatory factor analyses, internal consistency and validity assessment, and correlational analyses.

The second sample was recruited using the same procedure and the same inclusion and exclusion criteria as the first sample. The inclusion criteria were (a) being a currently enrolled university student in China, (b) being aged 18 years or older, (c) being able to read and complete the Chinese-language questionnaire, and (d) having prior experience using AI. The exclusion criteria were incomplete responses and response durations shorter than 60 s. This sample was reserved exclusively for assessing test–retest reliability. Thirty Chinese university students consented to participate and completed the IAIS scale at Time 1 (T1). After a 2-week interval, they completed it again at Time 2 (T2). All 30 participants provided valid responses at both time points, and no cases were excluded. The final valid sample consisted of 30 participants (*M*_age_ = 22.70 years, *SD* = 1.66; 50.0% male). Regarding educational attainment, 3 participants (10.0%) were vocational college students, 23 (76.7%) were undergraduates, and 4 (13.3%) were graduate students or above. Each participant received fixed monetary compensation upon completion.

### 2.2. Measures

#### 2.2.1. Inclusion of AI in Self

**Figure 1:**
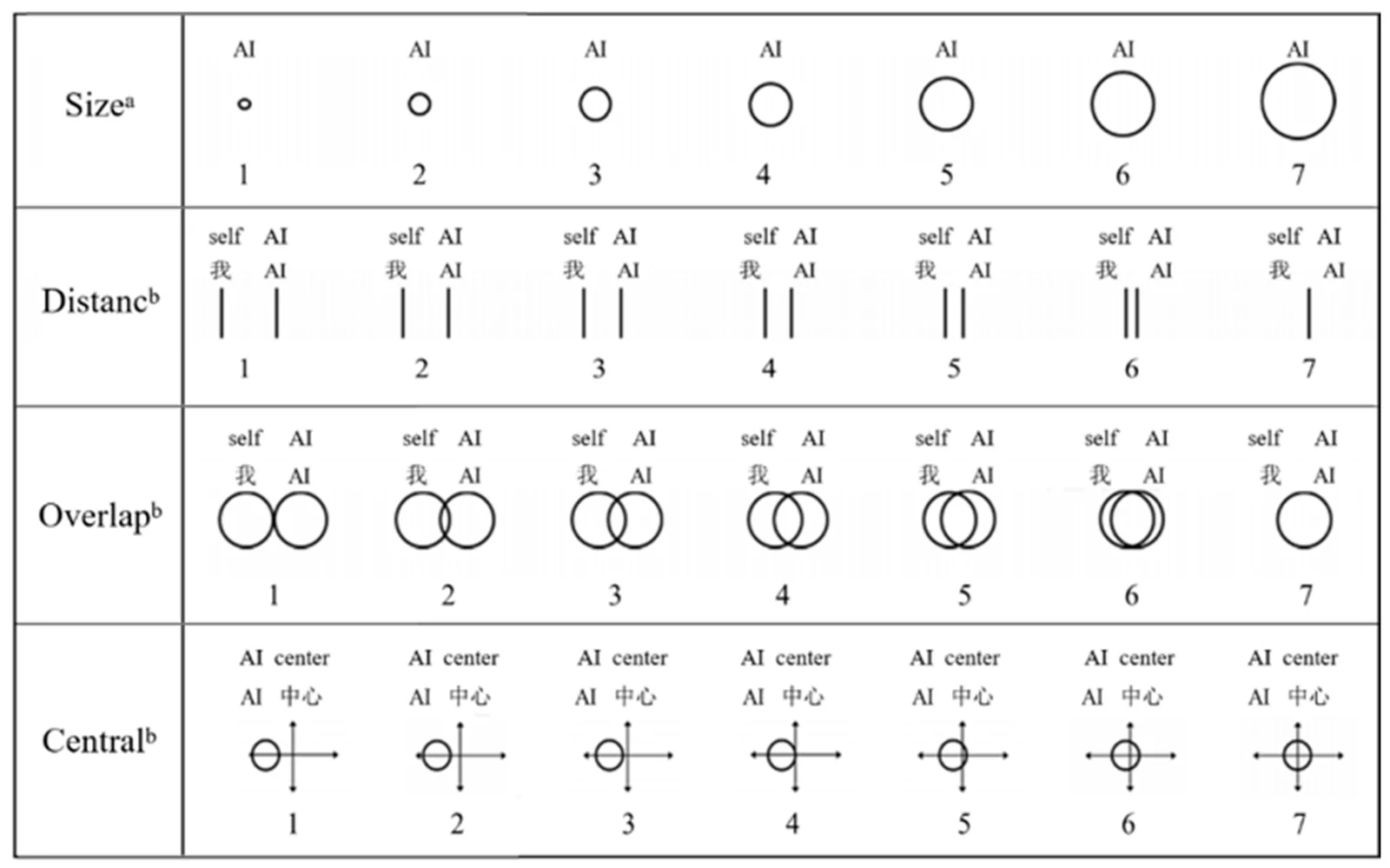
Inclusion of AI in Self scale. Note. Participants were first given IAIS instruction: “Choose the image that best describes your relationship with AI, answering with whatever first comes to mind.” Item-specific prompts then followed: (a) “Choose the image that best depicts AI as you consider your relationship with it”; (b) “Choose the image that best describes your relationship with AI.” The overlap item originated from the INS (Schultz, 2002); the other items originated from the EINS (Martin & Czellar, 2016).

The Extended Inclusion of Nature in Self (EINS) scale, widely used in environmental psychology, employs a spatial metaphor framework to assess nature connectedness (Martin & Czellar, 2016). The present study adapted its four items (overlap, distance, centrality, and size) to AI-mediated contexts to develop the IAIS scale. Specifically, “self” and “nature” in the original scale were replaced with “me” and “AI,” respectively. Through four pictorial items, the scale evaluates individuals’ perceived “degree of fusion between me and AI.” For example, in the “size” item, participants express their identification with AI by selecting the relative size of circles; in the “overlap” item, participants select from seven pairs of circles the diagram that best describes their “relationship with AI” (see Figure 1).

Seven experts in social psychology and human–AI interaction independently rated each of the four items on a 4-point scale (1 = not relevant/clear, 4 = highly relevant/clear) regarding (a) relevance to the construct “inclusion of AI in self” and (b) clarity/comprehensibility. Item-level content validity indices (I-CVI) and scale-level indices (S-CVI) were computed. The I-CVIs ranged from 0.86 to 1.00 for relevance and were all 1.00 for clarity; S-CVI/Ave was 0.98, and the modified kappa (K) of each item ranged from 0.85 to 1.00. All indices met the recommended criteria of I-CVI ≥ 0.78, S-CVI/Ave ≥ 0.90, and K ≥ 0.74 (Lynn, 1986; Polit & Beck, 2006; Polit et al., 2007), indicating that the scale had good content validity.

In addition, cognitive interviews with 10 participants were conducted to assess how the pictorial items were interpreted. Responses were independently coded by two trained coders, with high inter-coder agreement (Cohen’s κ = 0.84; 87.5% agreement). Their responses mapped onto the four metaphors in the theoretically intended way: the size item evoked AI’s significance and influence; the distance item evoked relational intimacy and accessibility; the overlap item evoked cognitive inclusion of AI in the self; and the centrality item evoked AI’s core position within the self. These responses indicate that respondents interpreted the four pictorial items as intended, corroborating the expert content-validity evidence.

#### 2.2.2. AI Identity

AI identity was measured using a nine-item scale adapted from the IT Identity Scale originally developed by Carter (2012) and later revised into a three-factor, nine-item version by P. Wang et al. (2022). Following Mirbabaie et al. (2022), the items were rephrased to refer to AI rather than general IT. The scale comprises three dimensions—emotional energy, relatedness, and dependence—each assessed by three items (e.g., “Thinking about myself in relation to AI, I feel energized”). Responses were recorded on a 5-point Likert scale (1 = strongly disagree, 5 = strongly agree). Cronbach’s α in the present study was 0.82.

#### 2.2.3. AI Attachment

The Experiences in Human-AI Relationships Scale (EHARS) developed by Yang and Oshio (2025) was employed to measure individuals’ attachment relationships with AI systems. This scale comprises 7 items (e.g., “I often ask AI to show more feeling and affection.”) rated on a 7-point Likert scale (1 = strongly disagree, 7 = strongly agree). In the present study, the scale demonstrated a Cronbach’s alpha of 0.82.

#### 2.2.4. AI Literacy

The AI Literacy Scale (AILS) developed by B. Wang et al. (2023) was employed to assess participants’ AI literacy. This scale comprises 12 items (e.g., “I am always alert to the abuse of AI technology.”) rated on a 7-point Likert scale (1 = strongly disagree, 7 = strongly agree). In the present study, the scale demonstrated a Cronbach’s alpha of 0.77.

#### 2.2.5. AI Self-Efficacy

The General Self-Efficacy Scale of Artificial Intelligence (GSE-6AI) developed by Y.-Y. Wang and Chuang (2024) was employed to assess participants’ AI self-efficacy. This scale comprises 6 items (e.g., “If someone opposes me, I can find means and ways to get what I want by using artificial intelligence”) rated on a 4-point Likert scale (1 = not at all true, 4 = exactly true). In the present study, the scale yielded a Cronbach’s alpha of 0.72.

#### 2.2.6. AI Dependence

The Artificial Intelligence Chatbot Dependence Scale (AICDS; Zhang et al., 2025) was employed to measure individuals’ dependence on AI chatbots. The original scale comprises 8 items rated on a 7-point Likert scale (1 = strongly disagree, 7 = strongly agree). In the present study, the abbreviated 6-item version validated by Çakmak et al. (2026) in a Turkish context was adopted (e.g., “I find it difficult to control the amount of time I spend using AI chatbots”). This refined version demonstrated good internal consistency in the current sample, with a Cronbach’s alpha of 0.88.

### 2.3. Data Analysis

Descriptive statistics, reliability analyses, and exploratory factor analysis (EFA) were conducted in SPSS 26.0; confirmatory factor analysis (CFA) and multigroup CFA were conducted in AMOS 28.0. For the factor analyses, the subsample sizes (*n* = 97 for EFA, *n* = 98 for CFA) satisfied the recommended participant-to-item ratio for a four-item scale but fell slightly below the N ≥ 100 minimum commonly recommended for CFA; KMO values further supported sampling adequacy. Because these samples are modest, we treat the factor-structure estimates as preliminary and in need of replication in larger samples.

First, Sample 1 was randomly split into two groups. One group (*n* = 97) was used for EFA to explore the latent structure of IAIS (Costello & Osborne, 2005); the other group (*n* = 98) was used for CFA to test the fit of the proposed factor structure. Sampling adequacy was assessed using the Kaiser–Meyer–Olkin (KMO) measure and Bartlett’s test of sphericity. A KMO value greater than 0.70 indicates that the data are suitable for factor analysis (Kaiser, 1974). The number of factors to retain was determined with Horn’s (1965) parallel analysis, in which the empirical eigenvalues were compared against the eigenvalues obtained from random data. The model fit indices included the chi-square-to-degrees-of-freedom ratio (χ^2^/*df* < 3), comparative fit index (CFI > 0.95), Tucker–Lewis index (TLI > 0.95), root mean square error of approximation (RMSEA ≤ 0.06), and standardized root mean square residual (SRMR < 0.08) (Hu & Bentler, 1998). In addition, average variance extracted (AVE) was obtained from CFA output. Convergent validity is supported when the items of each factor share a high proportion of variance and the within-factor correlations are significant. According to established criteria, good convergent validity is indicated when AVE > 0.50 and composite reliability (CR) > 0.70 (Hair et al., 2011).

Second, internal consistency reliability and test–retest reliability were examined. Cronbach’s α was used to evaluate internal consistency, whereas test–retest reliability involved administering the scale to participants at two time points separated by two weeks to assess response consistency over time. Additionally, item discrimination was examined by computing item–total correlations and by comparing respondents in the upper and lower 27% of total scores. This analysis indicates how well each item distinguishes between participants with high versus low levels of the construct.

Third, to examine the discriminant validity of IAIS relative to the criterion variables, we used two approaches: the Fornell–Larcker criterion and the heterotrait–monotrait ratio (HTMT). The Fornell–Larcker criterion requires that the square root of AVE for each construct exceed its correlations with other constructs (Fornell & Larcker, 1981). The HTMT ratio assesses construct distinctiveness by comparing the mean of correlations between items measuring different constructs with the mean of correlations between items measuring the same construct; HTMT < 0.85 indicates good discriminant validity (Henseler et al., 2015).

Fourth, to compare the correlations of IAIS and the single-item measure with the criterion variables, we used Lee and Preacher’s (2013) online calculator for comparing dependent correlations. Based on Steiger’s (1980) Z test, this tool tests whether two correlations that share a variable (e.g., the correlations of IAIS and of the single-item measure with the same criterion) differ significantly. This analysis was used to evaluate the criterion-related validity of IAIS relative to the single-item measure. To further rule out the possibility that the IAIS outperforms the single-item measure merely because aggregation yields higher reliability, hierarchical regression analyses were conducted with each criterion regressed first on the single-item measure and then on IAIS; the change in *R*^2^ indexed the incremental criterion-related validity of the four spatial metaphors (Hunsley & Meyer, 2003; Diamantopoulos et al., 2012).

Finally, to test whether IAIS had equivalent measurement properties across male and female respondents, multigroup confirmatory factor analysis (MGCFA) was conducted to sequentially test configural invariance, metric invariance, and scalar invariance. Model comparisons used ΔCFI ≤ 0.01 as the primary criterion, supplemented by Δ*χ*^2^ tests for reference (Cheung & Rensvold, 2002).

### 2.4. Results

#### 2.4.1. Exploratory Factor Analysis

For the EFA, Bartlett’s test of sphericity was significant, *χ*^2^ = 148.80, *df* = 6, *p* < .001, and KMO was 0.77, indicating that the data were suitable for factor analysis. The EFA supported a unidimensional structure: size (λ_1_ = 0.73), distance (λ_2_ = 0.85), overlap (λ_3_ = 0.83), and centrality (λ_4_ = 0.85) all loaded on a single common factor, which explained 66.50% of the item variance. Horn’s (1965) parallel analysis supported the unidimensional solution: only the first empirical eigenvalue (2.60) exceeded the corresponding eigenvalue obtained from random data (1.16), and both the mean and 95th-percentile criteria indicated a single factor.

#### 2.4.2. Confirmatory Factor Analysis

The CFA showed good model fit: *χ*^2^ = 0.81, *df* = 2, *p* = .668, RMSEA = 0.00, SRMR = 0.01, TLI = 0.99, CFI = 1.00, IFI = 1.00, AIC = 16.81. Factor loadings ranged from 0.65 to 0.81, meeting the criteria for convergent validity. For the single-factor model, average variance extracted (AVE) was 0.52 and composite reliability (CR) was 0.81, meeting the thresholds of AVE > 0.50 and CR > 0.70 (Hair et al., 2011).

#### 2.4.3. Reliability Analysis

In Sample 1, Cronbach’s α was 0.82 and McDonald’s ω was 0.82. In Sample 2, Cronbach’s α was 0.81 at T1 and 0.84 at T2, and the intraclass correlation coefficient (ICC) was 0.88. IAIS scores at T1 (*M* = 5.23, *SD* = 0.77) and T2 (*M* = 5.27, *SD* = 0.72) were strongly correlated, *r* = 0.79, *p* < .001. These results indicate that IAIS has good reliability among university students.

#### 2.4.4. Item Discrimination Analysis

Inter-item correlations ranged from 0.49 to 0.63 (*M* = 0.53), all below 0.70, suggesting no substantial redundancy among items and that each item captures a somewhat distinct facet of the construct (Boyle, 1991). Item-total correlations ranged from 0.69 to 0.84, indicating high homogeneity between each item and the scale as a whole (see Table 2).

Using the upper- and lower-27% criterion, respondents were divided into high- and low-scoring groups based on their total scores. Results showed that size (*t* = −12.91, *p* < .001; Cohen’s d = 0.74), distance (*t* = −21.24, *p* < .001; Cohen’s d = 0.72), overlap (*t* = −26.35, *p* < .001; Cohen’s d = 0.57), and centrality (*t* = −18.06, *p* < .001; Cohen’s d = 0.74) all differed significantly between the high- and low-scoring groups. These results indicate good item discrimination.

#### 2.4.5. Discriminant Validity of IAIS Relative to Criterion Variables

Results of the Fornell–Larcker criterion indicated that the square root of AVE for IAIS (0.73) exceeded its latent correlations with AI identity (0.71), AI attachment (0.47), AI literacy (0.42), AI self-efficacy (0.63), and AI dependence (0.53), satisfying the requirements for discriminant validity. Furthermore, the HTMT matrix revealed that all HTMT values between IAIS and the criterion variables fell below the strict threshold of 0.85 (range = 0.24–0.58), further supporting the distinctiveness of IAIS from these adjacent constructs at the item level.

Because IAIS and AI identity were strongly correlated, we conducted supplementary χ^2^ difference tests to provide further evidence of discriminant validity. Constraining the correlation between IAIS and AI identity to unity significantly worsened model fit, Δ*χ*^2^_(1)_ = 79.89, *p* < .001, indicating that the latent correlation between the two constructs was significantly smaller than 1.00 (Anderson & Gerbing, 1988).

#### 2.4.6. Coefficient Difference Tests

IAIS (*M* = 5.10, *SD* = 0.88) and the single-item measure (*M* = 4.80, *SD* = 1.24) were highly positively correlated (*r* = 0.82, *p* < .001), suggesting that both instruments tap the same core construct. IAIS was significantly correlated with all criterion variables (*p* < .01). The two instruments’ correlations with the criteria differed significantly for all criterion variables except AI literacy (see Table 3).

Criterion-related validity tests showed that IAIS was more strongly correlated with most criterion variables than was the single-item measure. Specifically, the correlations of IAIS with AI identity (*Z* = −4.21, *p* < .001), AI self-efficacy (*Z* = −2.63, *p* = .009), AI attachment (*Z* = −2.24, *p* = .025), and AI dependence (*Z* = −2.30, *p* = .021) were significantly higher than those of the single-item measure. For AI literacy, however, the advantage of IAIS was not significant. The correlation between IAIS and AI literacy (*r* = 0.20, *p* = .006) did not differ significantly from that of the single-item measure (*r* = 0.13, *p* = .064; *Z* = −1.64, *p* = .100).

To assess incremental validity, each criterion was regressed on the single-item measure and then on IAIS. For AI identity, the single-item measure explained 20.2% of the variance, *F*_(1, 193)_ = 48.82, *p* < .001, and IAIS increased the explained variance to 36.5%, Δ*R*^2^ = 0.16, Δ*F*_(3, 190)_ = 16.24, *p* < .001. Significant increments were likewise obtained for AI self-efficacy (*R*^2^ = 0.16 to 0.26, Δ*R*^2^ = 0.10, Δ*F* = 8.57, *p* < .001), AI dependence (*R*^2^ = 0.13 to 0.21, Δ*R*^2^ = 0.08, Δ*F* = 6.32, *p* < .001), and AI attachment (*R*^2^ = 0.09 to 0.16, Δ*R*^2^ = 0.07, Δ*F* = 5.01, *p* = .002). For AI literacy, the increment was small but significant (*R*^2^ = 0.02 to 0.06, Δ*R*^2^ = 0.04, Δ*F* = 2.78, *p* = .042), and the overlap item did not significantly predict AI literacy, *F*_(1, 193)_ = 3.47, *p* = .064, consistent with the Z-test results above.

#### 2.4.7. Cross-Gender Stability Analysis

Results of configural invariance tests indicated that the unrestricted multigroup baseline model fit well, *χ*^2^_(4)_ = 4.20, *p* = .380, RMSEA = 0.02, SRMR = 0.02, TLI = 1.00, CFI = 1.00, suggesting that the single-factor structure of IAIS was equivalent in form across male and female groups. Constraining factor loadings to be equal across groups to test metric invariance also produced acceptable fit, *χ*^2^_(7)_ = 8.93, *p* = .257, RMSEA = 0.04, SRMR = 0.03, TLI = 0.99, CFI = 0.99, with Δ*χ*^2^_(3)_ = 4.73, *p* = .192, ΔCFI = 0.01, meeting the 0.01 cutoff criterion, indicating that the contribution of each item to the latent variable was equivalent across genders. Building on the metric model, intercepts were further constrained to be equal across groups to test scalar invariance, which continued to fit well, *χ*^2^_(10)_ = 12.42, *p* = .258, RMSEA = 0.04, SRMR = 0.04, TLI = 0.99, CFI = 0.99, yielding Δ*χ*^2^_(3)_ = 3.49, *p* = .323, ΔCFI < 0.01, still below the cutoff. These results indicate that IAIS satisfied measurement invariance at the configural, metric, and scalar levels, supporting legitimate comparisons of latent variable means across gender groups (Cheung & Rensvold, 2002).

## 3. Study 2

Study 2 extended the validation context to organizational settings, examining the applicability of IAIS among employees, for whom AI use may be more concentrated and tool dependence higher. To streamline measurement, Study 2 employed only AI identity, AI literacy, and AI self-efficacy as validity criteria and tested whether the advantages of IAIS over the single-item measure replicated in a large sample.

### 3.1. Participants and Procedure

As in Study 1, Study 2 involved two independent samples. The inclusion criteria for the first sample were (a) being currently employed in China, (b) being aged 18 years or older, (c) being able to read and complete the Chinese-language questionnaire, and (d) having prior experience using AI. The first sample was recruited via the Credamo research platform; 520 Chinese employees volunteered to participate. After excluding seven cases with insufficient response durations (<60 s), the final dataset comprised 513 participants (*M*_age_ = 33.16, *SD* = 9.30; 38.4% male). Of these, 129 (25.2%) were from the state-owned sector, 330 (64.3%) from the private sector, and 54 (10.5%) from other organizations. Upon completion, each participant received a fixed monetary reward.

The second sample was recruited using the same procedure and the same inclusion and exclusion criteria as the first sample. The inclusion criteria were (a) being currently employed in China, (b) being aged 18 years or older, (c) being able to read and complete the Chinese-language questionnaire, and (d) having prior experience using AI. The exclusion criteria were incomplete responses and response durations shorter than 60 s. This sample was reserved exclusively for assessing test–retest reliability. Thirty Chinese employees consented to participate and completed the IAIS scale at Time 1 (T1). After a 2-week interval, they completed it again at Time 2 (T2). All 30 participants provided valid responses at both time points, and no cases were excluded. The final valid sample consisted of 30 participants (*M*_age_ = 33.57, *SD* = 9.56; 36.7% male), including 11 (36.7%) from the state-owned sector, 16 (53.3%) from the private sector, and 3 (10.0%) from other organizations. Each participant received a fixed monetary reward upon completion.

### 3.2. Measures

Study 2 used the same scales as Study 1 for IAIS, AI identity, AI literacy, and AI self-efficacy. Cronbach’s alphas were 0.74 for AI identity, 0.82 for AI literacy, and 0.70 for AI self-efficacy.

### 3.3. Results

#### 3.3.1. Exploratory Factor Analysis

For the EFA (*n* = 256), Bartlett’s test of sphericity was significant, *χ*^2^ = 346.40, *df* = 6, *p* < .001, and KMO was 0.80, indicating that the data were suitable for factor analysis. The EFA supported a unidimensional structure: size (λ_1_ = 0.72), distance (λ_2_ = 0.84), overlap (λ_3_ = 0.83), and centrality (λ_4_ = 0.82) all loaded on a single common factor, which explained 64.63% of the item variance. Horn’s (1965) parallel analysis likewise indicated a single factor: only the first empirical eigenvalue (2.60) exceeded the random-data counterpart (1.10) under both the mean and 95th-percentile criteria.

#### 3.3.2. Confirmatory Factor Analysis

To test whether the unidimensional structure identified in Study 1 replicated in this independent employee sample, we conducted a CFA (*n* = 257). The model fit well: *χ*^2^ = 1.09, *df* = 2, *p* = .579, RMSEA = 0.00, SRMR = 0.01, TLI = 1.00, CFI = 1.00, IFI = 1.00, AIC = 17.09. Factor loadings ranged from 0.58 to 0.92, meeting the criteria for convergent validity. For the single-factor model, average variance extracted (AVE) was 0.56 and composite reliability (CR) was 0.83, meeting the thresholds of AVE > 0.50 and CR > 0.70 (Hair et al., 2011).

#### 3.3.3. Reliability Analysis

In Sample 1, Cronbach’s α was 0.82 and McDonald’s ω was 0.82. In Sample 2, Cronbach’s α was 0.81 at T1 and 0.85 at T2, and the intraclass correlation coefficient (ICC) was 0.90. IAIS scores at T1 (*M* = 5.42, *SD* = 0.68) and T2 (*M* = 5.51, *SD* = 0.69) were strongly correlated, *r* = 0.83, *p* < .001. These results indicate that the IAIS has good reliability among employees.

#### 3.3.4. Item Discrimination Analysis

Inter-item correlations ranged from 0.43 to 0.66 (*M* = 0.53), all below 0.70, suggesting no substantial redundancy among items and that each item captures a somewhat distinct facet of the construct (Boyle, 1991). Item-total correlations ranged from 0.69 to 0.87, indicating high homogeneity between each item and the scale as a whole (see Table 4).

Using the upper- and lower-27% criterion, respondents were divided into high- and low-scoring groups based on their total scores. Results showed that size (*t* = −23.24, *p* < .001; Cohen’s d = 0.59), distance (*t* = −29.56, *p* < .001; Cohen’s d = 0.69), overlap (*t* = −42.53, *p* < .001; Cohen’s d = 0.53), and centrality (*t* = −23.84, *p* < .001; Cohen’s d = 0.84) all differed significantly between the high- and low-scoring groups. These results indicate good item discrimination.

#### 3.3.5. Discriminant Validity of IAIS Relative to Criterion Variables

In Study 2, discriminant validity was supported for AI literacy and AI self-efficacy and partially supported for AI identity. The Fornell–Larcker criterion indicated that the square root of AVE for IAIS (0.74) exceeded its latent correlations with AI literacy (0.59) and AI self-efficacy (0.61). The latent correlation between IAIS and AI identity (0.81), however, slightly exceeded the square root of AVE, so the Fornell–Larcker criterion was not met for this pair, although the HTMT ratio (0.65) fell below the strict threshold of 0.85. Constraining the correlation between IAIS and AI identity to unity again significantly worsened model fit, Δ*χ*^2^_(1)_ = 129.66, *p* < .001, indicating that the latent correlation between the two constructs was significantly smaller than 1.00.

#### 3.3.6. Coefficient Difference Tests

IAIS (*M* = 5.26, *SD* = 0.84) and the single-item measure (*M* = 4.88, *SD* = 1.12) were highly positively correlated (*r* = 0.87, *p* < .001), suggesting that both instruments tap the same core construct. IAIS was significantly correlated with all criterion variables (*p* < .001) (see Table 5).

Criterion-related validity tests showed that IAIS was more strongly correlated with all three criterion variables than was the single-item measure. Specifically, the correlations of IAIS with AI identity (*Z* = −4.19, *p* < .001), AI literacy (*Z* = −3.88, *p* < .001), and AI self-efficacy (*Z* = −2.00, *p* = .045) were significantly higher than those of the single-item measure (the overlap item).

As in Study 1, each criterion was regressed first on the overlap item (the single-item measure) and then on IAIS. For AI identity, the single-item measure explained 36.9% of the variance, *F*_(1, 511)_ = 298.24, *p* < .001, and IAIS increased the explained variance to 46.6%, Δ*R*^2^ = 0.10, Δ*F*_(3, 508)_ = 30.76, *p* < .001. Significant increments were also obtained for AI literacy (*R*^2^ = 0.13 to 0.19, Δ*R*^2^ = 0.07, Δ*F* = 13.57, *p* < .001) and AI self-efficacy (*R*^2^ = 0.18 to 0.23, Δ*R*^2^ = 0.04, Δ*F* = 9.36, *p* < .001).

#### 3.3.7. Cross-Gender Stability Analysis

Results of configural invariance tests indicated that the unrestricted multigroup baseline model fit well, *χ*^2^_(4)_ = 4.19, *p* = .380, RMSEA = 0.01, SRMR = 0.01, TLI = 1.00, CFI = 1.00, suggesting that the single-factor structure of IAIS was equivalent in form across male and female groups. Constraining factor loadings to be equal across groups to test metric invariance also produced excellent fit, *χ*^2^_(7)_ = 4.65, *p* = .703, RMSEA = 0.00, SRMR = 0.02, TLI = 1.00, CFI = 1.00, with Δ*χ*^2^_(3)_ = 0.46, *p* = .929, ΔCFI = 0.00, meeting the 0.01 cutoff criterion, indicating that the contribution of each item to the latent variable was equivalent across genders. Building upon metric invariance, intercepts were further constrained to be equal across groups to test scalar invariance, which continued to fit well, *χ*^2^_(10)_ = 5.56, *p* = .851, RMSEA = 0.00, SRMR = 0.02, TLI = 1.00, CFI = 1.00, yielding Δ*χ*^2^_(3)_ = 0.91, *p* = .824, ΔCFI < 0.01, still below the cutoff. These results indicate that IAIS satisfied measurement invariance at the configural, metric, and scalar levels, supporting legitimate comparisons of latent variable means across gender groups (Cheung & Rensvold, 2002).

#### 3.3.8. Student–Employee Stability Analysis

Results of configural invariance tests indicated that the unrestricted multigroup baseline model fit well, *χ*^2^_(4)_ = 4.19, *p* = .382, RMSEA = 0.01, SRMR = 0.01, TLI = 1.00, CFI = 1.00, suggesting that the single-factor structure of IAIS was equivalent in form across student and employee groups. Constraining factor loadings to be equal across groups to test metric invariance also produced excellent fit, *χ*^2^_(7)_ = 8.63, *p* = .280, RMSEA = 0.02, SRMR = 0.02, TLI = 1.00, CFI = 1.00, with Δ*χ*^2^_(3)_ = 4.44, *p* = .217, ΔCFI = 0.00, meeting the 0.01 cutoff criterion, indicating that the contribution of each item to the latent variable was equivalent across groups. Building upon metric invariance, intercepts were further constrained to be equal across groups to test scalar invariance, which continued to fit well, *χ*^2^_(10)_ = 17.11, *p* = .072, RMSEA = 0.03, SRMR = 0.03, TLI = 0.99, CFI = 0.99, yielding Δ*χ*^2^_(3)_ = 8.48, *p* = .037, ΔCFI < 0.01, still below the cutoff. Although the Δ*χ*^2^ test was nominally significant, this statistic is highly sensitive to sample size and tends to reject even substantively negligible misspecifications in large samples (Chen, 2007); consistent with the criterion adopted in the present study, we therefore evaluated invariance primarily on the basis of ΔCFI ≤ 0.01 (Cheung & Rensvold, 2002), which remained within the threshold. These results indicate that IAIS satisfied measurement invariance at the configural, metric, and scalar levels, supporting legitimate comparisons of latent variable means across student and employee groups.

## 4. Study 3

Study 3 employed a two-wave longitudinal design with a 15-day follow-up to examine the moderating role of IAIS in the relationship between AI identity and AI threat and thereby to clarify the theoretical value of IAIS as a boundary condition beyond AI identity.

### 4.1. Participants and Procedure

Study 3 used a two-wave online design with an interval of approximately 15 days. A G*Power 3.1 analysis (medium effect size f^2^ = 0.15, α = 0.05, power = 0.80) indicated a minimum required sample size of approximately 109 participants (Cohen, 1988). The inclusion criteria were (a) being either a currently enrolled university student or a currently employed worker in China, (b) being aged 18 years or older, (c) being able to read and complete the Chinese-language questionnaire, and (d) having prior experience using AI. We recruited both Chinese university students and employees through the Credamo platform. At Time 1 (T1), participants completed the IAIS and AI identity scale, and reported demographic information (e.g., gender, age, and role type) and daily AI usage duration. About 15 days later, we sent them a follow-up link to measure AI threat perception. A total of 293 participants completed the T1 survey. After excluding incomplete questionnaires and those with response times <60 s, 280 participants remained. At Time 2 (T2), we further excluded participants who did not complete the follow-up survey, provided incomplete responses, or had response times <60 s. In addition, one case failed the attention checks and was dropped. The final paired sample included 240 participants (*M*_age_ = 27.98, *SD* = 8.59; 43.3% male), comprising 121 university students and 119 employees.

### 4.2. Measures

AI identity and IAIS were measured with the same scales as in Study 1 (Cronbach’s α = 0.82 and 0.75, respectively).

To measure AI threat, we used a four-item abbreviated scale developed by Brougham and Haar (2018) and refined by X. Li (2025). A sample item is “I worry that AI will replace my job one day.” Participants responded on a 5-point scale from 1 (not at all worried) to 5 (extremely worried), with higher scores reflecting greater perceived threat. Reliability was acceptable (Cronbach’s α = 0.77).

### 4.3. Data Analysis

Data were analyzed with SPSS 26.0 and the PROCESS macro (version 4.1; Hayes, 2022).

First, to preliminarily examine the direct effect of AI identity on AI threat, a linear regression model (Model 1) was constructed, with T2 AI threat as the dependent variable, T1 AI identity as the independent variable, and gender, age, role, and daily AI usage duration as covariates. Second, to examine the incremental effect of IAIS, a main-effect model (Model 2) was constructed by adding T1 IAIS as an additional predictor to Model 1, without including any interaction term. Third, a simple moderation model (Model 3) was estimated using PROCESS Model 1, with T2 AI threat as the dependent variable, T1 AI identity as the independent variable, T1 IAIS as the moderator, and gender, age, role, and daily AI usage duration as covariates. The continuous predictors (T1 AI identity and T1 IAIS) were mean-centered. The bootstrap method (5000 resamples) was used to estimate the 95% confidence interval for the interaction effect. Simple slope analyses were further conducted to examine the conditional effects of AI identity on AI threat at different levels of IAIS (±1 SD). A Johnson–Neyman analysis was conducted to identify the regions of significance for the IAIS. Finally, to test whether the moderating effect of IAIS on the relationship between AI identity and AI threat was further moderated by role, a three-way moderation model (Model 4) was estimated using the PROCESS Model 3 macro. In this model, T2 AI threat was the dependent variable, T1 AI identity was the independent variable, T1 IAIS was the first moderator, and role was the second moderator. Role was mean-centered before analysis. Gender, age, and daily AI usage duration were included as covariates. The model included all two-way interactions (AI identity × IAIS, AI identity × role, and IAIS × role) and the three-way interaction (AI identity × IAIS × role). The bootstrap method (5000 resamples) was used to estimate 95% confidence intervals for the interaction effects.

### 4.4. Results

#### 4.4.1. Confirmatory Factor Analysis

The CFA yielded *χ*^2^ = 7.33, *df* = 2, *p* = .026, RMSEA = 0.10, SRMR = 0.03, TLI = 0.93, CFI = 0.98, IFI = 0.98, and AIC = 23.33. Although the *χ*^2^ test was significant and the RMSEA exceeded conventional cutoff values, these indices should be interpreted cautiously given the very small degrees of freedom of the present model. In low-*df* models, RMSEA is known to perform poorly and may substantially overstate model misfit, even when the model is correctly or closely specified (Kenny et al., 2015). Accordingly, greater weight can be placed on complementary fit indices that are less affected by small degrees of freedom, particularly the CFI and SRMR (Shi et al., 2021). In the present analysis, the CFI (0.98), IFI (0.98), and SRMR (0.03) all indicated good fit, while the TLI (0.93) was somewhat below the conventional 0.95 criterion. Taken together, the pattern of fit indices suggests that the single-factor model can be accepted.

Standardized factor loadings ranged from 0.52 to 0.76, and composite reliability was acceptable (CR = 0.76), exceeding the recommended 0.70 threshold (Hair et al., 2011). The average variance extracted (AVE = 0.45) was slightly below the commonly recommended 0.50 benchmark. However, given that all standardized loadings exceeded 0.50 and CR exceeded 0.70, the results provide reasonable, albeit not strong, support for convergent validity (Fornell & Larcker, 1981).

#### 4.4.2. Descriptive Statistics and Correlation Analysis

Descriptive statistics and zero-order correlations among the Study 3 variables are presented in Table 6. AI identity was moderately and positively correlated with IAIS (*r* = 0.58, *p* < .001). Potential multicollinearity was examined further in the regression analyses using variance inflation factors and tolerance statistics.

#### 4.4.3. Moderating Effect Test

In Model 1, the overall model was statistically significant, *R*^2^ = 0.08, *F*_(5, 234)_ = 4.30, *p* < .001, and all VIF values were below 2 (tolerance ≥ 0.54), indicating no serious multicollinearity. AI identity had a significant direct effect on AI threat (*b* = −0.37, *SE* = 0.11, *p* = .001, 95% CI [−0.60, −0.18]).

In Model 2, the overall model was statistically significant, *R*^2^ = 0.15, *F*_(6, 233)_ = 6.74, *p* < .001. Adding IAIS increased the explained variance by approximately 7% (Δ*R*^2^ = 0.07), and all VIF values were below 2 (tolerance ≥ 0.54), indicating no serious multicollinearity. The direct effect of AI identity on AI threat was nonsignificant (*b* = −0.05, *SE* = 0.13, *p* = .722, 95% CI [−0.29, 0.21]), whereas the direct effect of IAIS on AI threat was significant (*b* = −0.37, *SE* = 0.09, *p* < .001, 95% CI [−0.55, −0.19]).

In Model 3, the overall model was statistically significant, *R*^2^ = 0.19, *F*_(7, 232)_ = 7.62, *p* < .001. Adding the AI identity × IAIS interaction increased the explained variance by approximately 4% (Δ*R*^2^ = 0.04), and all VIF values were below 2 (tolerance ≥ 0.53), indicating no serious multicollinearity. The direct effect of AI identity on AI threat was nonsignificant (*b* = −0.21, *SE* = 0.14, *p* = .140, 95% bootstrap CI [−0.47, 0.03]), whereas the direct effect of IAIS on AI threat was significant (*b* = −0.41, *SE* = 0.09, *p* < .001, 95% bootstrap CI [−0.59, −0.24]). More importantly, the interaction effect between AI identity and IAIS was significant (*b* = −0.41, *SE* = 0.12, *p* = .001, 95% bootstrap CI [−0.65, −0.16]), indicating that the association between AI identity and AI threat depended on the degree to which individuals incorporated AI into their self-representation (see Table 7).

**Figure 2:**
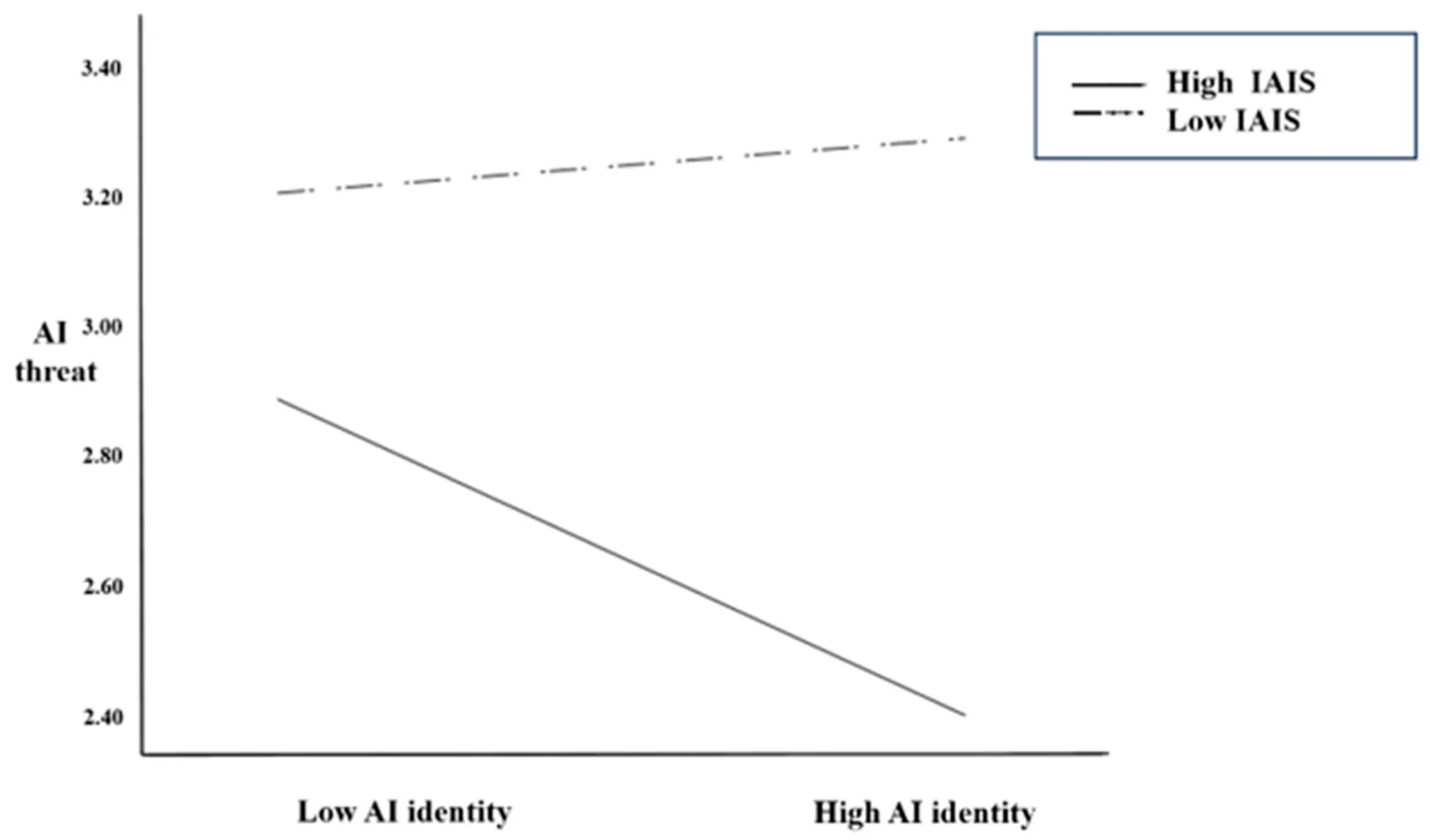
Simple slope analysis.

Simple slope analyses further revealed the direction and pattern of this moderating effect (see Figure 2). When IAIS was high (+1 SD), AI identity was significantly negatively associated with AI threat (*b* = −0.50, *SE* = 0.19, *p* = .009, 95% CI [−0.87, −0.13]); that is, higher identity was associated with lower threat perception. When IAIS was low (−1 SD), the association between AI identity and AI threat was nonsignificant (*b* = 0.09, *SE* = 0.14, *p* = .529, 95% CI [−0.18, 0.36]). These results indicate that IAIS serves as a boundary condition in the relationship between AI identity and AI threat.

A Johnson–Neyman analysis was conducted to further probe the significant interaction between AI identity and IAIS. The results identified two regions of significance. When centered IAIS was below −1.36, the conditional association between AI identity and AI threat was significantly positive; however, only 5.42% of the sample fell within this region. When centered IAIS exceeded 0.22, the association became significantly negative, with 51.67% of the sample falling above this threshold. Between these two values, the conditional effect was not statistically significant. Thus, at relatively high levels of IAIS, greater AI identity was associated with lower subsequent AI threat.

Finally, to test whether the effect of AI identity on AI threat was moderated by IAIS and role, a PROCESS Model 3 analysis was conducted with role mean-centered. The overall model was significant, *R*^2^ = 0.20, *F*_(10, 229)_ = 5.70, *p* < .001. The highest-order interaction, AI identity × IAIS × role, was not significant (*b* = 0.07, *SE* = 0.30, *t* = 0.22, *p* = .824, 95% bootstrap CI [−0.50, 0.71]), indicating that the moderating effect of IAIS on the relationship between AI identity and AI threat did not significantly vary by role. The two-way interaction AI identity × IAIS was significant (*b* = −0.37, *SE* = 0.15, *t* = −2.51, *p* = .013, 95% bootstrap CI [−0.70, −0.10]), whereas AI identity × role and IAIS × role were not significant (*ps* > .05). IAIS had a significant negative main effect on AI threat (*b* = −0.39, *SE* = 0.09, *t* = −4.30, *p* < .001, 95% bootstrap CI [−0.57, −0.21]). AI identity and role main effects, as well as all covariates, were not significant. Bootstrap results with 5000 resamples were consistent with these findings. Overall, the results support a significant AI identity × IAIS interaction but do not support a three-way moderation by role.

## 5. Discussion

Grounded in self-expansion theory and the AI-MC model, this study developed the IAIS scale and evaluated it with exploratory and confirmatory factor analyses, dependent correlation comparisons, and a two-wave moderation analysis. Results indicated that, in both university student and employee samples, the four spatial-metaphor items (overlap, distance, centrality, and size) loaded on a single latent factor, with good item discrimination, scale reliability, convergent validity, and criterion-related validity, supporting H1 and H2. The study also showed that IAIS correlated more strongly with the criteria (AI identity, AI self-efficacy, AI attachment, and AI dependence) than did the single-item measure. For AI literacy, the advantage of IAIS was significant in the employee sample but not in the student sample. Finally, IAIS moderated the relationship between AI identity and AI threat, supporting H3 and offering a possible account of the inconsistent prior evidence on this relationship.

### 5.1. The Structure of IAIS

Exploratory and confirmatory factor analyses showed that the four spatial-metaphor items of IAIS (overlap, distance, centrality, and size) loaded onto a single common factor, indicating that IAIS is a unidimensional construct. However, IAIS is not simply a replication of the classic IOS logic in the AI context; rather, it is a psychological structure that can be reliably measured within the framework of an expandable self-boundary. Specifically, the single-item measure assesses self–AI fusion solely through the “overlap” metaphor, which makes it difficult to capture the uniqueness of AI as a cognitive niche (Heersmink, 2020). Unlike the IOS pictorial measure, IAIS integrates multiple spatial metaphors, simultaneously reflecting boundary fusion (overlap), psychological proximity (distance), identity importance (size), and identity centrality (centrality). This structure is intended to cover the functional, ontological, and anthropomorphic pathways through which AI may reshape self-representation (Campbell et al., 2025), providing a more theoretically grounded operationalization.

This finding extends to human–AI interaction the mechanism posited in self-expansion theory, whereby individuals enhance their potential efficacy by drawing on external resources. It is consistent with the view that AI can serve as an external cognitive resource incorporated into the self-system (Aron et al., 1991; Aron et al., 2022; Clark & Chalmers, 1998). Specifically, the incorporation of AI into the self is not reducible to intimacy or anthropomorphic attachment; rather, it appears to reflect a dynamic process in which individuals integrate AI’s cognitive resources, decision-making capabilities, informational scaffolding, and identity-related value into their self-concept. This account is consistent with the extended mind thesis regarding cognitively distributed extension (Clark, 2007; Clark & Chalmers, 1998) and aligns with symbiotic technology theory’s emphasis on bidirectional human–AI coordination and dynamic equilibrium as defining features of the human–AI relationship (Van Lu, 2025).

The gender invariance observed in both samples indicates that the IAIS measures the same construct in men and women. Gendered socialization may well produce divergent AI usage patterns or attitudes (Stöhr et al., 2024), but it does not appear to alter the basic cognitive architecture through which individuals incorporate AI into self-concept. This also implies that the four spatial metaphors are interpreted equivalently across gender groups, supporting the use of IAIS as a general rather than gender-specific indicator.

### 5.2. Associations Between IAIS and Validity Criteria

The incremental value of IAIS over the single-item measure lies not simply in containing more items, but in providing broader content coverage of the same underlying construct of inclusion of AI in self. Whereas the single-item measure captures this construct primarily through perceived self–AI overlap, IAIS assesses it through four complementary spatial representations—overlap, distance, centrality, and size—thereby capturing a wider range of ways in which AI may be psychologically represented as part of the self. Steiger’s Z tests showed that IAIS correlated significantly more strongly with AI identity, AI self-efficacy, AI attachment, and AI dependence than did the single-item measure. Such findings indicate that IAIS captures criterion-relevant variance that may be insufficiently represented by an overlap-only item, thereby supporting its incremental measurement value relative to the single-item approach. Moreover, although students and employees may differ in their goals for using AI (Pyrkosz-Pacyna et al., 2022), IAIS showed good criterion-related validity in both samples, suggesting that its core psychological representation is similar across groups, much as the EINS showed that its spatial metaphors are represented by a unified construct (Martin & Czellar, 2016).

However, only in the employee sample did IAIS show a significantly stronger link to AI literacy than the single-item measure; among students, the difference was not significant. This population-specific pattern may reflect differences in usage context and capability development; however, these contextual factors were not directly measured, and the cross-sectional design cannot establish their causal role. For employees, AI use is often woven into work performance through complex problem-solving, decision support, and knowledge integration (Ishaq et al., 2025). In such contexts, AI literacy encompasses not merely operational skills but also metacognitive evaluation and strategic deployment of AI capabilities—namely, understanding how AI extends one’s professional competence and occupational identity (Carter & Grover, 2015). IAIS captures the deep cognitive and functional integration between AI and the self-concept through its four complementary metaphors (overlap, distance, centrality, and size), which aligns with the conception of AI literacy as understanding AI capability boundaries and effectively integrating them into workflow (Long & Magerko, 2020). By contrast, a single-item pictorial measure is less able to distinguish between “instrumental use” and “self-concept extension”; thus, in the employee sample, the four-item IAIS more sensitively reflects the association between AI literacy and self-extension than the single-item measure.

Among students, AI use tends to be concentrated on relatively standardized academic tasks such as information retrieval, assisted writing, and improving learning efficiency (Castillo-Martínez et al., 2024), in which AI literacy may primarily reflect basic operational proficiency and information discernment rather than self-reconstruction at the level of occupational identity. In this context, the association between AI and the self may remain at the level of a “tool” without extending to a structural transformation of the self-concept. A single-item measure may be sufficient to capture this relatively superficial perception of “self–AI overlap,” so the multi-metaphor advantage of IAIS was not evident in its association with AI literacy. Additionally, as university students are in a period of occupational identity exploration, their AI self-extension may be highly context-dependent and unstable, which may weaken the association between AI literacy and self-extension (Arnett, 2000).

### 5.3. The Moderating Effect of IAIS

The study found that IAIS significantly moderated the association between AI identity and AI threat, such that the negative association between AI identity and threat emerged only at relatively high levels of IAIS. This association disappeared when AI remained at the periphery of the self and, at very low levels of IAIS, even became positive. According to self-expansion theory, a high level of IAIS reflects a process in which AI is cognitively assimilated into the self-concept through representational overlap, making the cognitive boundaries between self and AI permeable (Aron et al., 1991; Aron & Fraley, 1999). When AI is psychologically included in the self, people may be less likely to treat its autonomous capacities and decision-making resources as substitutes for human agency. Instead, these resources may be experienced as extensions of the self-system (Clark & Chalmers, 1998; Clark, 2008). Under these conditions, AI-assisted autonomy may be interpreted as a shared expansion of human–AI capabilities rather than as an incursion into one’s professional domain, which is consistent with lower threat appraisals (Mirbabaie et al., 2022; Petriglieri, 2011).

By contrast, when IAIS scores are low, people may show emotional investment in or behavioral reliance on AI without the representational overlap that marks genuine inclusion of other in the self (Aron et al., 1991; Aron & Fraley, 1999). Because AI remains psychologically external, its autonomous capacities may be attributed to an outside agent that appears to diminish human control over work-related domains. Without the assimilative processes that would include AI in the self, such autonomy is more likely to be appraised as encroaching on areas tied to professional expertise and occupational identity, consistent with heightened AI threat (Mirbabaie et al., 2022; Petriglieri, 2011).

These findings may help explain inconsistent evidence on the link between AI identity and AI threat. Earlier work suggests that stronger AI identity is associated with lower AI threat (Mirbabaie et al., 2022), but a more recent study reports a nonsignificant direct effect of AI identity on professional identity threat (Ashraf et al., 2026). We suggest that this inconsistency may stem from a measurement limitation: prior indices of AI identity do not distinguish among its underlying psychological forms. At low levels of IAIS, AI identity may primarily index superficial emotional attachment and shows little protective association with threat; at high levels of IAIS, AI identity may reflect deeper psychological integration, and its negative association with threat becomes significant. This pattern is consistent with recent meta-analytic evidence that human–AI collaboration can activate both opportunity- and threat-related processes, with the overall outcome depending on how individuals appraise the system (Song et al., 2026). IAIS may capture this appraisal process, offering a boundary condition that helps explain when AI identity is negatively associated with threat and when it may instead be positively associated with it. Moreover, this moderation pattern did not differ significantly between students and employees, suggesting that it holds across both groups.

### 5.4. Limitations and Contributions

This study has several limitations. First, the samples consisted mainly of university students and employees. Because participants were recruited through online platforms and were digitally engaged, they may have been more familiar with AI than the general population, which introduces possible sampling and platform-related biases. Future research should therefore test the scale’s structural stability across broader populations, including different age groups and freelancers.

Second, the study relied primarily on cross-sectional surveys, which limits conclusions about the dynamic development of the inclusion of AI in self. Moreover, although Study 3 employed a two-wave design, AI threat was measured only at Time 2 without a Time 1 baseline; accordingly, the Study 3 finding is interpreted as a temporally lagged association rather than as evidence of change in threat over time, and future research should include a baseline measure of AI threat to examine such change.

Third, the exploratory and confirmatory factor analyses in Study 1 were conducted on relatively small subsamples. Although the resulting unidimensional structure was subsequently supported in a larger independent employee sample (*N* = 513) in Study 2, the Study 1 estimates of factor loadings and model fit should nonetheless be considered preliminary rather than definitive. In addition, the test–retest reliability was assessed with only 30 participants in each population, which provides limited precision for claims about temporal stability. Accordingly, the present results provide initial rather than conclusive evidence for the validity of IAIS, and future research should replicate these findings in larger and more diverse samples to establish the stability and generalizability of the factor structure and to examine test–retest reliability and longitudinal measurement invariance.

Fourth, the scale was developed and validated in a single cultural setting (China), which limits its external validity. Concepts such as selfhood, psychological distance, identity centrality, dependence, and human–technology boundaries are not culturally neutral: in individualistic cultures, inclusion of AI in self may be interpreted in terms of autonomy, personal enhancement, and individual identity, whereas in relational or collectivist contexts it may be understood through interdependence, role performance, and socially embedded identity (Markus & Kitayama, 1991). Cross-cultural differences could therefore affect not only mean IAIS scores but also the meaning of the pictorial items themselves, consistent with the notion of a cultural cognition gap in human–AI interaction. Future validation should test measurement invariance across cultural and linguistic groups to establish the generalizability of the construct.

Fifth, the practical applications of the scale (e.g., AI-literacy education, human–AI collaboration, and dependence-risk monitoring) remain preliminary, and their generalizability across cultures, AI-use contexts, specific AI systems, and levels of AI experience awaits further validation. In particular, as security and privacy concerns surrounding large language models grow (e.g., backdoor threats) and as these models are increasingly used in program analysis (J. Wang et al., 2025; Zhou et al., 2025), the scale’s applicability across these emerging AI contexts warrants further examination. In addition, a latent-variable moderation model would account for measurement error but is under-identified for a four-item scale; examining latent moderation with a longer instrument is a direction for future research.

The theoretical contributions are threefold. First, this study extends the scope of self-expansion theory. By extending the IOS mechanism from traditional domains such as intimate relationships, nature connection, and group identity to human–AI interaction in digital contexts, this study suggests that individuals can incorporate nonhuman, functional, distributed AI systems into the self-concept, extending the applicability of self-expansion theory in the digital era (Aron et al., 1991, 2022; Belk, 2013). Second, the study integrates multiple theoretical perspectives on AI self-expansion. Drawing on extended mind, cognitive scaffolding, digital extended self, and symbiotic technology theories, it proposes that IAIS is a unified process involving functional extension, cognitive integration, and identity embedding, moving beyond single frameworks that treat AI either as a mere tool or as an anthropomorphized companion (Buongiorno, 2019; Risko & Gilbert, 2016). Third, it provides a standardized measure of AI self-expansion, addressing the lack of psychometrically sound pictorial scales in this area and offering a replicable instrument for subsequent examination of its formation mechanisms, boundary conditions, and intervention strategies.

Building upon this foundation, future research can proceed in two directions. First, with the rise of generative AI companions (e.g., Replika, Character.AI), users can develop intimate experiences of “falling in love” and “being understood” through conversations with AI (H. Li & Zhang, 2024). The self-inclusion resulting from such quasi-intimate relationships may differ fundamentally from that produced by relationships with real partners. Specifically, self-expansion through real relationships, though accompanied by uncertainty, helps individuals accumulate experience in navigating complex interpersonal situations and enhances independence (Feeney, 2007), whereas the “secure” extension provided by AI companions may create a cognitive comfort zone, weakening individuals’ adaptive capacity to face real relationship conflicts (Kouros & Papa, 2024). Therefore, future research should track and compare the differential long-term effects of these two types of relational extension on individuals’ social functioning, relationship satisfaction, and mental health, so as to clarify the adaptive boundaries and potential risks of AI self-expansion. Second, from a developmental perspective, the temporal dynamics of AI self-extension warrant investigation. Self-expansion theory emphasizes that “novelty” and “challenge” in relationships are core drivers of extension (Aron & Aron, 1997). However, AI companions’ responses are shaped by training data and by reinforcement learning from human feedback (RLHF), including its sycophancy bias, which may erode users’ affective and cognitive trust by reducing perceived authenticity (Mu-En Liu et al., 2026) and thereby attenuate extension effects over time. A longitudinal study found that, owing to novelty effects, users initially developed friendships with a social chatbot (Mitsuku), but as its behavior became predictable, their enjoyment of the interaction declined (Croes & Antheunis, 2021).

## 6. Conclusions

Grounded in self-expansion theory and the AI-MC model, this study extended the pictorial measurement paradigm of inclusion in the self from interpersonal and nature domains to human–AI interaction contexts, developing the IAIS scale and providing initial psychometric evidence for its validity. Across student and employee populations, the four spatial-metaphor items showed good discrimination and loaded on a single factor, and the scale showed good internal consistency and test–retest reliability. IAIS also showed stronger criterion-related validity than the single-item measure for most criteria, as well as measurement invariance across gender. Furthermore, IAIS moderated the relationship between AI identity and AI threat, offering a possible account of inconsistent prior evidence on this relationship. Theoretically, these findings extend self-expansion theory to human–AI interaction by suggesting that AI can become psychologically incorporated into the self as a form of cognitive and functional self-extension. Practically, IAIS offers a standardized measure of this incorporation that complements conventional indicators such as AI use and attitudes, with potential applications in AI-literacy education, human–AI collaboration, and dependence-risk monitoring. Cross-cultural applications, however, should remain cautious until measurement invariance is established.

## Figures and Tables

**Table 1:** Meaning of each item.

Item	IAIS
Overlap	The extent to which individuals cognitively include AI in their self-concept.
Distance	The subjective feeling of intimacy and accessibility with AI.
Centrality	The core position of AI in self.
Size	The significance and influence of AI in the individual’s mind.

**Table 2:** Pearson correlations among the four items (Study 1).

	*M*	*SD*	1	2	3	4	5
1 Size	5.52	0.94	-				
2 Distance	4.83	1.23	0.49 ***	-			
3 Overlap	4.80	1.24	0.49 ***	0.63 ***	-		
4 Centrality	5.17	1.16	0.50 ***	0.58 ***	0.52 ***	-	
5 IAIS	5.10	0.88	0.69 ***	0.84 ***	0.82 ***	0.82 ***	-

Note. ***, *p* < .001. *M*, mean value; *SD*, standard deviation.

**Table 3:** Descriptive Statistics, Correlation Analyses, and Coefficient Difference Tests (Study 1).

	*M*	*SD*	1	2	*Z*
1 single-item	4.80	1.24	-		
2 IAIS	5.10	0.88	0.82 ***	-	
3 AI Identity	4.11	0.49	0.45 ***	0.60 ***	−4.21 ***
4 AI Attachment	4.66	1.17	0.30 ***	0.39 ***	−2.24 *
5 AI Literacy	5.51	0.65	0.13	0.20 **	−1.64
6 AI self-efficacy	3.22	0.41	0.40 ***	0.50 ***	−2.63 **
7 AI Chatbot Dependence	4.91	1.18	0.36 ***	0.45 ***	−2.30 *

Note. ***, *p* < .001; **, *p* < .01; *, *p* < .05. *M*, mean value; *SD*, standard deviation.

**Table 4:** Pearson correlations among the four items (Study 2).

	*M*	*SD*	1	2	3	4	5
1 Size	5.60	0.81	-				
2 Distance	5.12	1.09	0.43 ***	-			
3 Overlap	4.88	1.12	0.50 ***	0.66 ***	-		
4 Centrality	5.45	1.14	0.43 ***	0.56 ***	0.60 ***	-	
5 IAIS	5.26	0.84	0.69 ***	0.84 ***	0.87 ***	0.82 ***	-

Note. ***, *p* < .001. *M*, mean value; *SD*, standard deviation.

**Table 5:** Descriptive Statistics, Correlation Analyses, and Coefficient Difference Tests (Study 2).

	*M*	*SD*	1	2	Z
1 single-item	4.88	1.12	-		
2 IAIS	5.26	0.84	0.87 ***	-	
3 AI Literacy	5.86	0.48	0.35 ***	0.43 ***	−3.88 ***
4 AI identity	4.15	0.48	0.61 ***	0.68 ***	−4.19 ***
5 AI self-efficacy	3.27	0.38	0.43 ***	0.47 ***	−2.00 *

Note. ***, *p* < .001; *, *p* < .05. *M*, mean value; *SD*, standard deviation.

**Table 6:** Descriptive statistics and Pearson correlations among study variables (Study 3).

	*M*	*SD*	1	2	3	4	5	6
1 AI identity	4.17	0.48	-					
2 IAIS	5.27	0.72	0.58 ***	-				
3 AI threat	2.86	0.82	−0.24 ***	−0.36 ***	-			
4 Age	27.98	8.59	0.15 *	0.17 *	−0.12	-		
5 Gender	1.57	0.50	−0.01	−0.05	0.13 *	0.11	-	
6 Use time	2.55	2.12	0.07	0.22 ***	−0.07	0.13	−0.05	-
7 Role	1.50	0.50	−0.12	0.03	0.01	0.62 ***	0.23 ***	0.11

Note. ***, *p* < .001; *, *p* < .05. Gender, 1 = male, 2 = female; role, 1 = student, 2 = employee. *M*, mean value; *SD*, standard deviation.

**Table 7:** Regression results for the moderating role of IAIS (Study 3).

Variables	Model 1				Model 2				Model 3			
	*b*	*SE*	*t*	95% CI	*b*	*SE*	*t*	95% CI	*b*	*SE*	*t*	95% CI ^a^
Age	−0.01	0.01	−1.38	[−0.03, 0.01]	−0.01	0.01	−1.37	[−0.03, 0.01]	−0.01	0.01	−0.94	[−0.02, 0.01]
Gender	0.22 *	0.11	2.08	[0.01, 0.43]	0.19	0.10	1.83	[−0.02, 0.40]	0.17	0.10	1.66	[−0.03, 0.37]
Use time	−0.01	0.03	−0.53	[−0.06, 0.04]	0.01	0.02	0.31	[−0.04, 0.06]	0.00	0.02	0.05	[−0.05, 0.07]
Role	0.05	0.14	0.33	[−0.23, 0.32]	0.09	0.14	0.68	[−0.18, 0.36]	0.13	0.13	0.95	[−0.13, 0.38]
AI identity	−0.37 **	0.11	−3.23	[−0.59, −0.14]	−0.05	0.13	−0.36	[−0.31, 0.22]	−0.21	0.14	−1.48	[−0.47, 0.03]
IAIS					−0.37 ***	0.09	−4.18	[−0.54, −0.19]	−0.41 ***	0.09	−4.72	[−0.59, −0.24]
AI identity × IAIS									−0.41 **	0.12	−3.34	[−0.65, −0.16]
Constant	2.78 ***	0.24	11.78	[2.32, 3.25]	2.70 ***	0.23	11.78	[2.25, 3.15]	2.68 ***	0.22	11.95	[2.21, 3.13]
*R* ^2^	0.08				0.15				0.19			
Δ*R*^2^					0.07				0.04			
*F*	4.30 ***				6.74 ***				7.62 ***			

Note. ***, *p* < .001; **, *p* < .01; *, *p* < .05. The dependent variable was AI threat. Role was coded as 1 = student, 2 = employee. ^a^, 95% confidence intervals were computed using 5000 bootstrap resamples.

## Data Availability

The data and study materials are available on OSF at https://osf.io/ba9hz/overview?view_only=6acc6f64782b4b108af882f1909338cf (accessed on 29 September 2026).

## Abbreviations

The following abbreviations are used in this manuscript:

AI	artificial intelligence
IAIS	inclusion of AI in self

## References

[B1-jintelligence-14-00239] Alabed A., Javornik A., Gregory-Smith D. (2022). AI anthropomorphism and its effect on users’ self-congruence and self–AI integration: A theoretical framework and research agenda. Technological Forecasting and Social Change.

[B2-jintelligence-14-00239] Allen M. S., Iliescu D., Greiff S. (2022). Single item measures in psychological science. European Journal of Psychological Assessment.

[B3-jintelligence-14-00239] Anderson J. C., Gerbing D. W. (1988). Structural equation modeling in practice: A review and recommended two-step approach. Psychological Bulletin.

[B4-jintelligence-14-00239] Arnett J. J. (2000). Emerging adulthood: A theory of development from the late teens through the twenties. American Psychologist.

[B5-jintelligence-14-00239] Aron A., Aron E. N. (1986). Love and the expansion of self: Understanding attraction and satisfaction.

[B6-jintelligence-14-00239] Aron A., Aron E. N. (1997). Self-expansion motivation and including other in the self. Handbook of personal relationships: Theory, research and interventions.

[B7-jintelligence-14-00239] Aron A., Aron E. N., Smollan D. (1992). Inclusion of other in the self scale and the structure of interpersonal closeness. Journal of Personality and Social Psychology.

[B8-jintelligence-14-00239] Aron A., Aron E. N., Tudor M., Nelson G. (1991). Close relationships as including other in the self. Journal of Personality and Social Psychology.

[B9-jintelligence-14-00239] Aron A., Fraley B. (1999). Relationship closeness as including other in the self: Cognitive underpinnings and measures. Social Cognition.

[B10-jintelligence-14-00239] Aron A., Lewandowski G., Branand B., Mashek D., Aron E. (2022). Self-expansion motivation and inclusion of others in self: An updated review. Journal of Social and Personal Relationships.

[B11-jintelligence-14-00239] Ashraf A., Min Q., Ashraf A. (2026). A moderated mediation model of AI-driven identity threats and employee cyberloafing: The role of AI-inclusive identity. European Journal of Investigation in Health, Psychology and Education.

[B12-jintelligence-14-00239] Belk R. W. (1988). Possessions and the extended self. Journal of Consumer Research.

[B13-jintelligence-14-00239] Belk R. W. (2013). Extended self in a digital world. Journal of Consumer Research.

[B14-jintelligence-14-00239] Bollen K., Lennox R. (1991). Conventional wisdom on measurement: A structural equation perspective. Psychological Bulletin.

[B15-jintelligence-14-00239] Boyle G. J. (1991). Does item homogeneity indicate internal consistency or item redundancy in psychometric scales?. Personality and Individual Differences.

[B16-jintelligence-14-00239] Brougham D., Haar J. (2018). Smart technology, artificial intelligence, robotics, and algorithms (STARA): Employees’ perceptions of our future workplace. Journal of Management & Organization.

[B17-jintelligence-14-00239] Bruner J. S., Postman L. (1958). Symbolic value as an organizing factor in perception. Understanding human motivation.

[B18-jintelligence-14-00239] Buongiorno F. (2019). From the extended mind to the digitally extended self: A phenomenological critique. Aisthesis. Pratiche, Linguaggi E Saperi dell’Estetico.

[B19-jintelligence-14-00239] Camacho L. J. (2026). The steered self: Algorithmic consumer identity theory in platformized markets. Businesses.

[B20-jintelligence-14-00239] Campbell S. W., Ellison N. B., Ross M. Q. (2025). Extending the self through AI-mediated communication: Functional, ontological, and anthropomorphic extensions. Communication and Change.

[B21-jintelligence-14-00239] Carter M. (2012). Information Technology (IT) identity: A conceptualization, proposed measures, and research agenda. Doctoral dissertation.

[B22-jintelligence-14-00239] Carter M., Grover V. (2015). Me, my self, and I(T): Conceptualizing information technology identity and ITS implications. MIS Quarterly.

[B23-jintelligence-14-00239] Castillo-Martínez I. M., Flores-Bueno D., Gómez-Puente S. M., Vite-León V. O. (2024). AI in higher education: A systematic literature review. Frontiers in Education.

[B24-jintelligence-14-00239] Chen F. F. (2007). Sensitivity of goodness of fit indexes to lack of measurement invariance. Structural Equation Modeling: A Multidisciplinary Journal.

[B25-jintelligence-14-00239] Cheung G. W., Rensvold R. B. (2002). Evaluating goodness-of-fit indexes for testing measurement invariance. Structural Equation Modeling.

[B26-jintelligence-14-00239] Clark A. (2007). Re-Inventing ourselves: The plasticity of embodiment, sensing, and mind. Journal of Medicine and Philosophy.

[B27-jintelligence-14-00239] Clark A. (2008). Supersizing the mind: Embodiment, action, and cognitive extension.

[B28-jintelligence-14-00239] Clark A. (2025). Extending minds with generative AI. Nature Communications.

[B29-jintelligence-14-00239] Clark A., Chalmers D. (1998). The extended mind. Analysis.

[B30-jintelligence-14-00239] Cohen J. (1988). Statistical power analysis for the behavioral sciences.

[B31-jintelligence-14-00239] Comrey A. L., Lee H. B. (1992). A first course in factor analysis.

[B32-jintelligence-14-00239] Costello A. B., Osborne J. (2005). Best practices in exploratory factor analysis: Four recommendations for getting the most from your analysis. Practical Assessment, Research & Evaluation.

[B33-jintelligence-14-00239] Croes E. A., Antheunis M. L. (2021). Can we be friends with mitsuku? A longitudinal study on the process of relationship formation between humans and a social chatbot. Journal of Social and Personal Relationships.

[B34-jintelligence-14-00239] Cronbach L. J. (1951). Coefficient alpha and the internal structure of tests. Psychometrika.

[B35-jintelligence-14-00239] Çakmak B., Özdemir E., Koç Ö., Doğutepe E. (2026). From prompts to dependency: Standardization of the AI chatbot dependence scale in a Turkish context and associations with big five personality traits. International Journal of Human–Computer Interaction.

[B36-jintelligence-14-00239] Diamantopoulos A., Sarstedt M., Fuchs C., Wilczynski P., Kaiser S. (2012). Guidelines for choosing between multi-item and single-item scales for construct measurement: A predictive validity perspective. Journal of the Academy of Marketing Science.

[B37-jintelligence-14-00239] Epley N., Waytz A., Cacioppo J. T. (2007). On seeing human: A three-factor theory of anthropomorphism. Psychological Review.

[B38-jintelligence-14-00239] Feeney B. C. (2007). The dependency paradox in close relationships: Accepting dependence promotes independence. Journal of Personality and Social Psychology.

[B39-jintelligence-14-00239] Folk D., Heine S. J., Dunn E. (2025). Individual differences in anthropomorphism help explain social connection to AI companions. Scientific Reports.

[B40-jintelligence-14-00239] Fornell C., Larcker D. F. (1981). Evaluating structural equation models with unobservable variables and measurement error. Journal of Marketing Research.

[B41-jintelligence-14-00239] Guingrich R. E., Graziano M. S. A. (2025). A longitudinal randomized control study of companion chatbot use: Anthropomorphism and its mediating role on social impacts. Proceedings of the AAAI/ACM Conference on AI, Ethics, and Society.

[B42-jintelligence-14-00239] Hair J. F., Ringle C. M., Sarstedt M. (2011). PLS-SEM: Indeed a silver bullet. Journal of Marketing Theory and Practice.

[B43-jintelligence-14-00239] Hayes A. F. (2022). Introduction to mediation, moderation, and conditional process analysis: A regression-based approach.

[B44-jintelligence-14-00239] Heersmink R. (2020). Varieties of the extended self. Consciousness and Cognition.

[B45-jintelligence-14-00239] Henseler J., Ringle C. M., Sarstedt M. (2015). A new criterion for assessing discriminant validity in variance-based structural equation modeling. Journal of the Academy of Marketing Science.

[B46-jintelligence-14-00239] Horn J. L. (1965). A rationale and test for the number of factors in factor analysis. Psychometrika.

[B47-jintelligence-14-00239] Hu L., Bentler P. M. (1998). Fit indices in covariance structure modeling: Sensitivity to underparameterized model misspecification. Psychological Methods.

[B48-jintelligence-14-00239] Huang B., Laporte S., Sénécal S., Sobol K. (2025). Falling out with AI-buddies: The hidden costs of treating AI as a partner versus servant during service failure. Technological Forecasting and Social Change.

[B49-jintelligence-14-00239] Hunsley J., Meyer G. J. (2003). The incremental validity of psychological testing and assessment: Conceptual, methodological, and statistical issues. Psychological Assessment.

[B50-jintelligence-14-00239] Hutchins E. (1995). Cognition in the wild.

[B51-jintelligence-14-00239] Iacobucci D. (2010). Structural equations modeling: Fit indices, sample size, and advanced topics. Journal of Consumer Psychology.

[B52-jintelligence-14-00239] Ishaq R., Gorsi H., Butt S., Umair T., Tajammal R. (2025). Beyond automation: Unleashing creativity and enhancing decision-making with generative AI. International Journal for Social Sciences.

[B53-jintelligence-14-00239] Jarvis C. B., MacKenzie S. B., Podsakoff P. M. (2003). A critical review of construct indicators and measurement model misspecification in marketing and consumer research. Journal of Consumer Research.

[B54-jintelligence-14-00239] Joseph J. (2025). The algorithmic self: How AI is reshaping human identity, introspection, and agency. Frontiers in Psychology.

[B55-jintelligence-14-00239] Kaiser H. F. (1974). An index of factorial simplicity. Psychometrika.

[B56-jintelligence-14-00239] Kenny D. A., Kaniskan B., McCoach D. B. (2015). The performance of RMSEA in models with small degrees of freedom. Sociological Methods & Research.

[B57-jintelligence-14-00239] Koo T. K., Li M. Y. (2016). A guideline of selecting and reporting intraclass correlation coefficients for reliability research. Journal of Chiropractic Medicine.

[B58-jintelligence-14-00239] Kouros T., Papa V. (2024). Digital mirrors: AI companions and the self. Societies.

[B59-jintelligence-14-00239] Lee I. A., Preacher K. J. (2013). Calculation for the test of the difference between two dependent correlations with one variable in common *[Computer software]*.

[B60-jintelligence-14-00239] Li H., Zhang R. (2024). Finding love in algorithms: Deciphering the emotional contexts of close encounters with AI chatbots. Journal of Computer-Mediated Communication.

[B61-jintelligence-14-00239] Li X. (2025). A study of the effect of perceived AI status threat on employee innovation performance: The moderating role of perceived benefits. Doctoral dissertation.

[B62-jintelligence-14-00239] Liang Q., Banks J. (2025). Perceived shared understanding between humans and artificial intelligence: Development and validation of a self-report scale. Technology, Mind, and Behavior.

[B63-jintelligence-14-00239] Long D., Magerko B. (2020). What is AI literacy? Competencies and design considerations. CHI ‘20: Proceedings of the 2020 CHI Conference on Human Factors in Computing Systems.

[B64-jintelligence-14-00239] Lynn M. R. (1986). Determination and quantification of content validity. Nursing Research.

[B65-jintelligence-14-00239] Markus H. R., Kitayama S., Strauss J., Goethals G. (1991). Cultural variation in the self-concept. The self: Interdisciplinary approaches.

[B66-jintelligence-14-00239] Martin C., Czellar S. (2016). The extended inclusion of nature in self scale. Journal of Environmental Psychology.

[B67-jintelligence-14-00239] Mashek D., Cannaday L. W., Tangney J. P. (2007). Inclusion of community in self scale: A single-item pictorial measure of community connectedness. Journal of Community Psychology.

[B68-jintelligence-14-00239] Mirbabaie M., Brünker F., Möllmann Frick N. R., Stieglitz S. (2022). The rise of artificial intelligence–understanding the AI identity threat at the workplace. Electronic Markets.

[B69-jintelligence-14-00239] Mu-En Liu J., Weng C. C. H., Hou Y. T. Y. (2026). When flattery backfires: How sycophancy and interaction context shape perceived authenticity and trust in large language AI models. Proceedings of the extended abstracts of the 2026 CHI conference on human factors in computing systems.

[B70-jintelligence-14-00239] Park C. S., Kaye B. K. (2019). Smartphone and self-extension: Functionally, anthropomorphically, and ontologically extending self via the smartphone. Mobile Media & Communication.

[B71-jintelligence-14-00239] Paullay I. M., Alliger G. M., Stone-Romero E. F. (1994). Construct validation of two instruments designed to measure job involvement and work centrality. Journal of Applied Psychology.

[B72-jintelligence-14-00239] Pentina I., Hancock T., Xie T. (2023). Exploring relationship development with social chatbots: A mixed-method study of replika. Computers in Human Behavior.

[B73-jintelligence-14-00239] Petriglieri J. L. (2011). Under threat: Responses to and the consequences of threats to individuals’ identities. Academy of Management Review.

[B74-jintelligence-14-00239] Polit D. F., Beck C. T. (2006). The content validity index: Are you sure you know what’s being reported? Critique and recommendations. Research in Nursing & Health.

[B75-jintelligence-14-00239] Polit D. F., Beck C. T., Owen S. V. (2007). Is the CVI an acceptable indicator of content validity? Appraisal and recommendations. Research in Nursing & Health.

[B76-jintelligence-14-00239] Pyrkosz-Pacyna J., Dukala K., Kosakowska-Berezecka N. (2022). Perception of work in the IT sector among men and women—A comparison between IT students and IT professionals. Frontiers in Psychology.

[B77-jintelligence-14-00239] Risko E. F., Gilbert S. J. (2016). Cognitive offloading. Trends in Cognitive Sciences.

[B78-jintelligence-14-00239] Schiller S. R., Signorelli C. M., Stamatiou F. (2025). The intercepted self: How generative AI challenges the dynamics of the relational self. Proceedings of the AAAI/ACM Conference on AI, Ethics, and Society.

[B79-jintelligence-14-00239] Schultz P. W., Schmuck P., Schultz W. P. (2002). Inclusion with nature: The psychology of human-nature relations. Psychology of sustainable development.

[B80-jintelligence-14-00239] Shi D., DiStefano C., Maydeu-Olivares A., Lee T. (2021). Evaluating SEM model fit with small degrees of freedom. Multivariate Behavioral Research.

[B81-jintelligence-14-00239] Skjuve M., Følstad A., Fostervold K. I., Brandtzaeg P. B. (2021). My chatbot companion—A study of human-chatbot relationships. International Journal of Human-Computer Studies.

[B82-jintelligence-14-00239] Song Y. X., Zeng M. Z., Su T. (2026). Opportunity or threat? A meta-analysis of the impact of human-AI collaboration systems on employee work effectiveness. Advances in Psychological Science.

[B83-jintelligence-14-00239] Steiger J. H. (1980). Tests for comparing elements of a correlation matrix. Psychological Bulletin.

[B84-jintelligence-14-00239] Stöhr C., Ou A. W., Malmström H. (2024). Perceptions and usage of AI chatbots among students in higher education across genders, academic levels and fields of study. Computers and Education: Artificial Intelligence.

[B85-jintelligence-14-00239] Tropp L. R., Wright S. C. (2001). Ingroup identification as the inclusion of ingroup in the self. Personality and Social Psychology Bulletin.

[B86-jintelligence-14-00239] Van Lu P. (2025). Symbiotic technology: The cognitive–technological architecture of homo symbioticus.

[B87-jintelligence-14-00239] Wang B., Rau P.-L. P., Yuan T. (2023). Measuring user competence in using artificial intelligence: Validity and reliability of artificial intelligence literacy scale. Behaviour & Information Technology.

[B88-jintelligence-14-00239] Wang J., Ni T., Lee W.-B., Zhao Q. (2025). A contemporary survey of large language model assisted program analysis. Transactions on Artificial Intelligence.

[B89-jintelligence-14-00239] Wang P., Liu H., Wang X., Yuan X., Sun Y., Xiao L., Li W., Ma C. (2022). A preliminary validation of the Chinese version of the information technology identity scale. Psychology Research and Behavior Management.

[B90-jintelligence-14-00239] Wang Y.-Y., Chuang Y.-W. (2024). Artificial intelligence self-efficacy: Scale development and validation. Education and Information Technologies.

[B91-jintelligence-14-00239] Williams L. E., Bargh J. A. (2008). Keeping one’s distance: The influence of spatial distance cues on affect and evaluation. Psychological Science.

[B92-jintelligence-14-00239] Yang F., Oshio A. (2025). Using attachment theory to conceptualize and measure the experiences in human-AI relationships. Current Psychology.

[B93-jintelligence-14-00239] Zhang X., Yin M., Zhang M., Li Z., Li H. (2025). The development and validation of an artificial intelligence chatbot dependence scale. Cyberpsychology, Behavior, and Social Networking.

[B94-jintelligence-14-00239] Zhou Y., Ni T., Lee W.-B., Zhao Q. (2025). A survey on backdoor threats in Large Language Models (LLMS): Attacks, defenses, and evaluation methods. Transactions on Artificial Intelligence.

